# Evolutionary impacts differ between two exploited populations of northern bottlenose whale (*Hyperoodon ampullatus*)

**DOI:** 10.1002/ece3.5813

**Published:** 2019-11-19

**Authors:** Laura Joan Feyrer, Paul Bentzen, Hal Whitehead, Ian G. Paterson, Anthony Einfeldt

**Affiliations:** ^1^ Biology Department Dalhousie University Halifax NS Canada

**Keywords:** demographic reconstruction, endangered species, low diversity, mitogenome, whaling

## Abstract

Interpretation of conservation status should be informed by an appreciation of genetic diversity, past demography, and overall trends in population size, which contribute to a species' evolutionary potential and resilience to genetic risks. Low genetic diversity can be symptomatic of rapid demographic declines and impose genetic risks to populations, but can also be maintained by natural processes. The northern bottlenose whale *Hyperoodon ampullatus* has the lowest known mitochondrial diversity of any cetacean and was intensely whaled in the Northwest Atlantic over the last century, but whether exploitation imposed genetic risks that could limit recovery is unknown. We sequenced full mitogenomes and genotyped 37 novel microsatellites for 128 individuals from known areas of abundance in the Scotian Shelf, Northern and Southern Labrador, Davis Strait, and Iceland, and a newly discovered group off Newfoundland. Despite low diversity and shared haplotypes across all regions, both markers supported the Endangered Scotian Shelf population as distinct from the combined northern regions. The genetic affinity of Newfoundland was uncertain, suggesting an area of mixing with no clear population distinction for the region. Demographic reconstruction using mitogenomes suggests that the northern region underwent population expansion following the last glacial maximum, but for the peripheral Scotian Shelf population, a stable demographic trend was followed by a drastic decline over a temporal scale consistent with increasing human activity in the Northwest Atlantic. Low connectivity between the Scotian Shelf and the rest of the Atlantic likely compounded the impact of intensive whaling for this species, potentially imposing genetic risks affecting recovery of this population. We highlight how the combination of historical environmental conditions and modern exploitation of this species has had very different evolutionary impacts on structured populations of northern bottlenose whales across the western North Atlantic.

## Graphical abstract

Distinct populations may respond differently to range‐wide exploitation, resulting in evolutionary impacts and reduced genetic diversity that can limit recovery and resilience of remnant populations. Sequencing the full mitogenomes and 37 novel microsatellites for 127 specimens of northern bottlenose whales (*Hyperoodon ampullatus*), we reconstructed the trajectories of two distinct populations that were heavily exploited by commercial whaling. We found that for a small population at their range edge, the impacts of whaling were more severe than for the larger more connected population, and combined with having the lowest range‐wide genetic variability of any cetacean, we suggest northern bottlenose whales may face genetic limitations to the recovery of their populations.

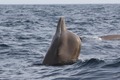

## 1 INTRODUCTION

Loss of genetic diversity can threaten the persistence of populations and species by reducing individual fitness (Amos & Balmford, 2001) and limiting their potential to adapt to environmental and ecological changes (Bürger & Lynch, 1995; Lacy, 1997; Willi, Van Buskirk, & Hoffmann, 2006). Where species have been subjected to intensive harvesting, experienced rapid demographic decline or habitat fragmentation due to human activity, extremely low levels of genetic diversity can be an indicator of impaired recovery (Hutchings, Butchart, Collen, Schwartz, & Waples, 2012) and increased risk of extinction (Frankham, 2005, 2015; Keller & Waller, 2002). However, populations that have not been through a recent bottleneck can also maintain low levels of genetic diversity through natural processes, such as climate regime shifts (Attard et al., 2015; De Bruyn et al., 2009; Westbury, Petersen, Garde, Heide‐Jørgensen, & Lorenzen, 2019), life history attributes (Romiguier et al., 2014), social structure (Whitehead, 1998), recurrent selective sweeps (Bazin, Glémin, & Galtier, 2006), and sexual selection (Amos & Harwood, 1998). Populations that have maintained low genetic diversity under equilibrium conditions are unlikely to harbor the same frequency of deleterious alleles as populations that have undergone recent genetic bottlenecks (Keller & Waller, 2002). Determining the cause of low genetic diversity in a population is crucial to understanding the genetic risks faced by species that have been subject to historical or ongoing anthropogenic impacts and informing management decisions that could determine their future persistence (Allendorf, 2017).

**Figure 1:**
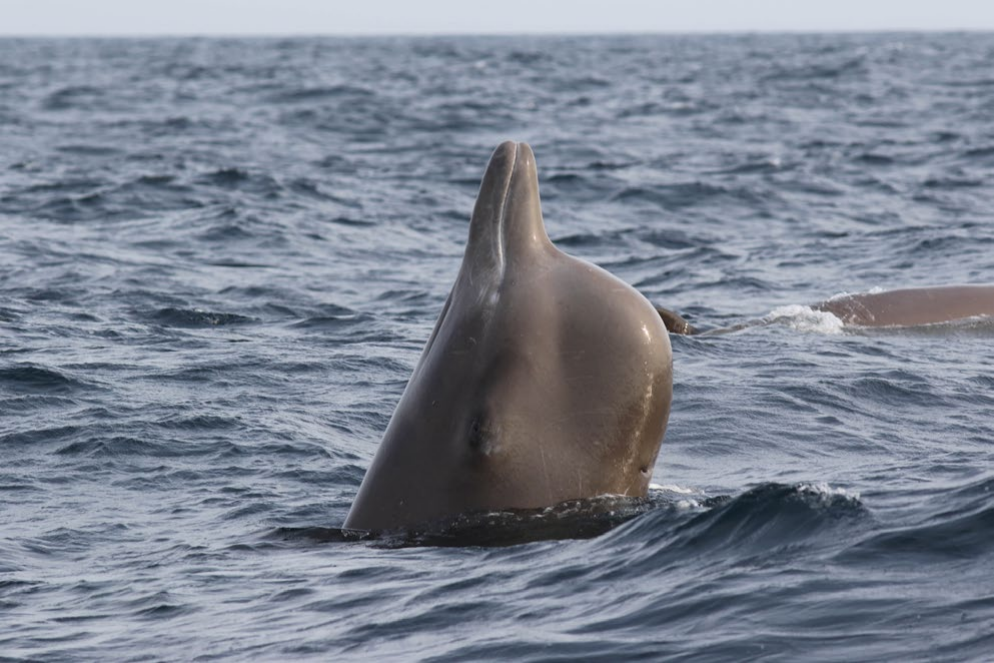
Photograph of northern bottlenose whale. (*Hyperoodon ampullatus) spy‐hopping in the Gully*,* Scotian Shelf*

The northern bottlenose whale (*Hyperoodon ampullatus*, Figure 1) currently has the lowest known mitochondrial diversity of any cetacean species (Whitehead, Vachon, & Frasier, 2017), but it is unknown whether this is due to recent declines from human harvesting or natural processes acting over longer time scales. *Hyperoodon ampullatus* was severely exploited over the course of the 19–20th centuries, and its current population size and recovery status are poorly understood. The range of *H. ampullatus* is restricted to the cold‐temperate North Atlantic, where approximately two‐thirds of the prewhaling population estimate of 65,000–100,000 whales were commercially whaled (Whitehead & Hooker, 2012). This level of exploitation likely resulted in a severe population decline (Christensen, 1973). Most of the early whaling effort was focused in core areas in the eastern north Atlantic, but in later years as catches declined whalers moved west, until commercial whaling of the species ended in 1971. Catch distributions suggest core whaling areas may reflect population structure, with subdivisions between the Scotian Shelf, the Labrador Sea, Iceland, Norway, and Svalbard (Benjaminsen, 1972; Whitehead & Hooker, 2012). If severe exploitation of *H. ampullatus* imposed a genetic bottleneck, population genetic theory predicts that deleterious alleles could increase in frequency, negatively impacting the recovery of their populations, especially where connectivity between core areas of abundance may be low (Keller & Waller, 2002; O'Grady et al., 2006).

While we know little about the other populations of northern bottlenose whales, the Scotian Shelf population has been the subject of long‐term field study and ongoing monitoring, and is currently listed as Endangered under Canada's *Species At Risk Act* due to small population size and isolation (COSEWIC, 2011; O'Brien & Whitehead, 2013; Whitehead, Faucher, Gowans, & McCarrey, 1997). This population is centered in the Gully, a submarine canyon and marine protected area (MPA). Between 1962 and 1971, commercial whaling took 87 whales from the Gully and more than 800 whales from the nearest known population off Northern Labrador. Reduced catch per unit effort across the North Atlantic suggests northern bottlenose whale populations were depleted when whaling ended in Canada in 1971 (Christensen, 1975; Mitchell, 1977; Reeves, Mitchell, & Whitehead, 1993; Whitehead & Hooker, 2012). Despite almost 50 years for population recovery, the most recent estimate from long‐term mark–recapture studies in the Gully indicates the Scotian Shelf population has remained small and stable at ~143 (CI = 129–156) from 1988 to 2011 (O'Brien & Whitehead, 2013). It is uncertain whether recovery has been limited by genetic, demographic, or ongoing anthropogenic factors (Whitehead & Hooker, 2012). Previous genetic analyses of 10 microsatellites and mitochondrial DNA control region sequences found genetic subdivision between the Scotian Shelf and the Northern Labrador Sea regions, but no evidence of genetic bottlenecks (Dalebout, Hooker, & Christensen, 2001; Dalebout, Ruzzante, Whitehead, & ØIen, 2006). However, the extremely low genetic diversity detected in *H. ampullatus* by Dalebout et al. (2006), particularly at mitochondrial control region sequences (5 haplotypes differentiated by 4 polymorphic sites in 127 individuals), provided limited power to resolve recent or historical demographic changes. Whether exploitation has contributed to the extraordinarily low genetic diversity or the population structure of *H. ampullatus* is therefore unclear.

There are a number of life history and selective processes that can sustain low levels of intraspecific genetic diversity over evolutionary time scales, predating major commercial exploitation efforts (Attard et al., 2015; Carroll et al., 2019; Ellegren & Galtier, 2016; Vachon, Whitehead, & Frasier, 2018). However, the patterns seen in *H. ampullatus* are not well explained by life history attributes commonly associated with naturally low genetic variation. Large body sizes, long generation times, and life spans have been associated with low genetic diversity in animals (Romiguier et al., 2014). Although *H. ampullatus* can grow larger than the average length found across all species of cetaceans, their body size is less than a third of the largest cetacean species, and their generation time is average compared with empirical and modeled estimates of age at first reproduction across cetacean species (Christensen, 1973; Taylor, Chivers, Larese, & Perrin, 2007). Sexual selection can lead to higher variance in reproductive success among males, which has been shown to reduce nuclear diversity relative to neutral expectations (Ellegren & Galtier, 2016; Wilson Sayres, 2018). Male sexual dimorphism is prevalent across Ziphiidae, and sexual selection has been widely accepted as an explanation for why they are the most diverse family of cetaceans (Dalebout, Steel, & Baker, 2008; Gol'din, 2014). However, it is not known how levels of nuclear diversity in *H. ampullatus* compare to other species and sexual selection does not explain the low mitochondrial diversity within *H. ampullatus* relative to other Ziphiidae. Male sexual dimorphism is prevalent across Ziphiidae, and sexual selection has been widely accepted as an explanation for why they are the most diverse family of cetaceans (Dalebout et al., 2008; Gol'din, 2014), but this does not explain the low diversity within *H. ampullatus* relative to other Ziphiidae. Cultural selection has been identified as a mechanism that can reduce mitochondrial diversity in matrilineal whales through cultural hitchhiking (Whitehead, 1998; Whitehead et al., 2017), and though *H. ampullatus* live in social groups, they form short‐term “fission–fusion” associations rather than long‐term matrilineal structures associated with cultural hitchhiking (Gowans, Whitehead, & Hooker, 2001). Recurrent selective sweeps for adaptive traits can reduce genetic diversity at mitochondrial loci (Bazin et al., 2006; Morin et al., 2018) and have previously been considered as a potential cause of low diversity in sperm whales (*Physeter macrocephalus*, Morin et al., 2018), killer whales (*Orcinus orca*, Foote et al., 2016), and false killer whales (*Pseudorca crassidens*, Martien et al., 2014). Some authors have suggested that deep diving, a trait shared across Ziphiidae, may be an adaptation that is under positive selection in cetaceans; however, other beaked whale species, *Ziphius cavirostris* (Dalebout et al., 2005) and *Mesoplodon mirus* (Thompson, Patel, Baker, Constantine, & Millar, 2016), do not appear to share the same low diversity as *H. ampullatus*. Our current understanding of the biology of *H. ampullatus* is limited and does not provide a clear explanation for low observed diversity.

Species with smaller population sizes are expected to have lower genetic diversity due to increased genetic drift (Leffler et al., 2012), and while population sizes are poorly understood in cetaceans, current population estimates for *H. ampullatus* are larger than at least five other species of cetaceans with higher measures of range‐wide mitochondrial nucleotide diversity (IUCN, 2018; Vachon et al., 2018). Historical demographic fluctuations can have lasting impacts on genetic diversity, and population dynamics associated with glacial oscillations are known to have had a major influence on patterns of genetic diversity in subpolar and temperate species (Hewitt, 2000). During the last glacial maximum (LGM) ~19–26 kya, the sea level was lower and ice sheets expanded toward the equator, resulting in genetic bottlenecks for many species including cetaceans in the northern (Jenkins, Castilho, & Stevens, 2018; Moura et al., 2014; Phillips et al., 2013) and southern hemispheres (Attard et al., 2015). In the North Atlantic, ice sheets covered a large portion of the current distribution of *H. ampullatus* (Paul & Schäfer‐Neth, 2003). This likely reduced available habitat for *H. ampullatus*, possibly limiting their population size and shifting their range southwards, followed by a population expansion as available habitat increased upon glacial recession. The last glacial maximum may have disproportionately affected *H. ampullatus* relative to other cetacean species due to a large portion of their shelf edge habitat being inaccessible and their specialization for deep water prey, primarily squid from the genus *Gonatus* (Hooker, Iverson, Ostrom, & Smith, 2001). Climatic fluctuations, foraging preferences, and a limited polar distribution relative to other species of beaked whales may have reduced available habitat and constrained overall population size, contributing to the low mitochondrial genetic diversity currently found in *H. ampullatus*.

Here, we investigate whether the extremely low mitochondrial diversity in the northern bottlenose whale results from genetic bottlenecks associated with intensive whaling or historical demographic changes during the last glacial maximum. Using a large panel of newly developed microsatellite markers and whole mitochondrial genomes, we first resolve population structure sampled across the Scotian Shelf, Labrador Sea, Davis Strait, Iceland, and Newfoundland. The specimens from Newfoundland represent the first observations of *H. ampullatus* in an area between the two known population centers of the Scotian Shelf and Labrador–Davis Strait, a region that has not, to our knowledge, been previously described in whaling records or scientific surveys. We examine the origin of the Newfoundland whales to assess whether they may represent an unexploited population, mixing between previously described subdivided populations, or signify the potential recovery and expansion of one of the core populations. We use whole mitochondrial genomes to reconstruct the historical demography of *H. ampullatus* and assess whether there is evidence of recent or historical genetic bottlenecks in the evolutionary trajectories of subpopulations. This represents the first population genetics study of northern bottlenose whales using mitogenomes and a large number of microsatellites from contemporary samples collected across the western North Atlantic.

## 2 MATERIALS AND METHODS

### 2.1 Sample collection

**Figure 2:**
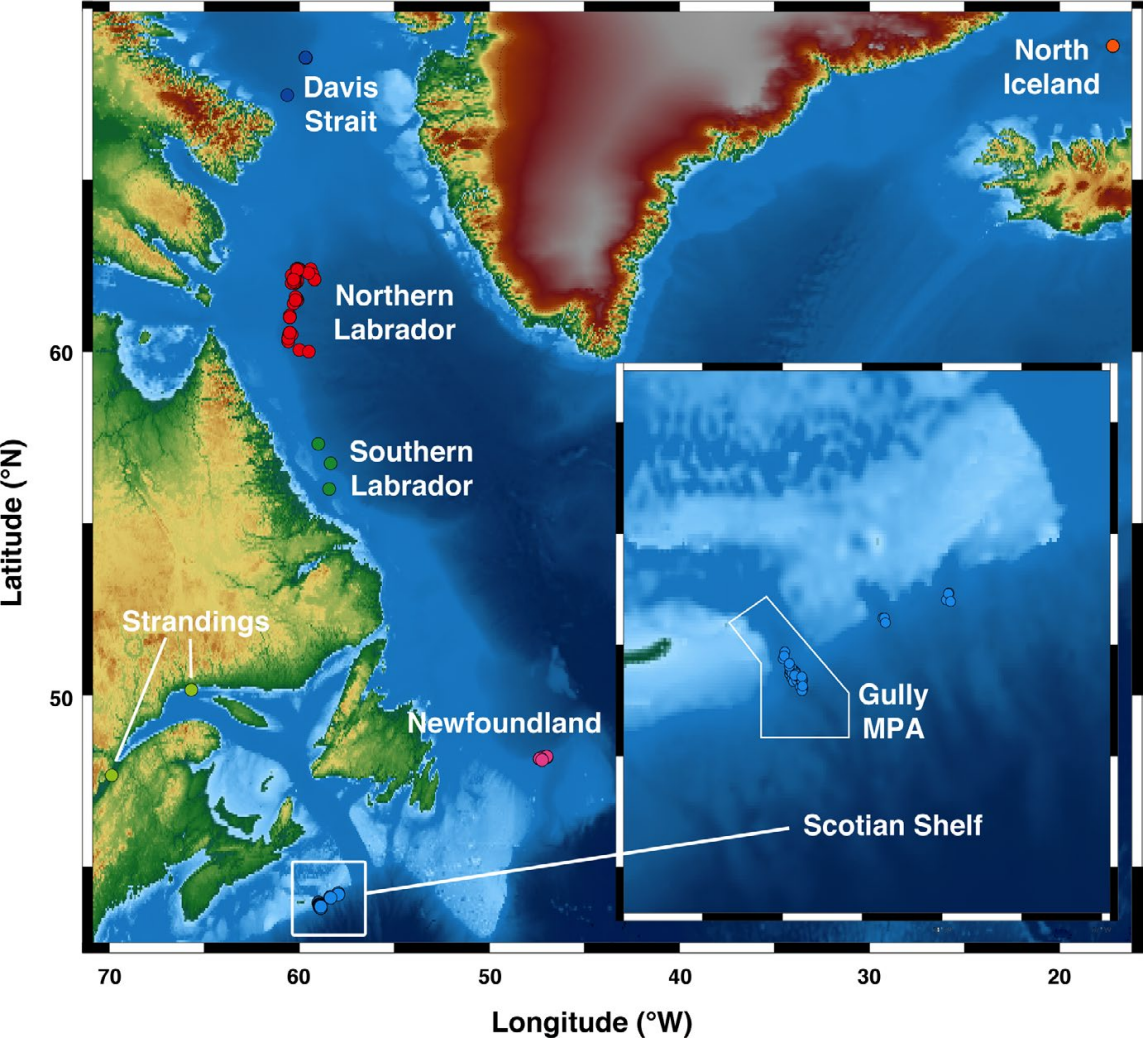
Location map of successfully sequenced samples across the study area, with inset of the Scotian Shelf. Circles indicate a sample; colors indicate population: Red—Northern Labrador, green—Southern Labrador, pink—Newfoundland, orange—Iceland, light blue—Scotian Shelf, dark blue—Davis Strait, and light green—Stranding

Initial samples were collected from 167 northern bottlenose whales (77 females, 90 males) from six locations in the North Atlantic: the Davis Strait, Northern Labrador Sea, Southern Labrador Sea, northern Iceland, the Scotian Shelf, and Newfoundland (Figure 2). Tissues sampled included dried gum tissue scraped from archived teeth collected during whaling more than 45 years ago (1967–1971), from biopsy samples collected 1997–2018 and samples collected from stranded whales around the region between 1994–2005. The sampling protocol for biopsy collection was reviewed and approved by the Dalhousie University Committee on Laboratory Animals and collected under permit from Canada's Department of Fisheries and Oceans (DFO). This study considers additional samples from the Scotian Shelf, a new sampling region in Newfoundland, and contemporary samples from the Davis Strait, that have not been included in any previous population analyses.

Northern Labrador samples were obtained from dried gum tissue collected from the teeth of 80 whales killed by whalers in the Northern Labrador Sea in 1971 (Christensen, 1973). Ten Davis Strait biopsy samples were collected opportunistically during Department of Fisheries and Oceans research cruises in 2017 and 2018. Three biopsy tissue samples collected in Southern Labrador in 2003 by Dalebout et al. (2006) were reanalyzed. For North Iceland, dried gum tissue was obtained from seven whales killed in the Norwegian hunt in 1967 (Benjaminsen, 1972). Gum tissue from whaling samples was collected as per Dalebout et al. (2006). For the Scotian Shelf samples, 60 biopsies of 54 unique individuals were obtained from free‐swimming whales in the summers of 1996, 1997, 2002, 2003, 2013, 2015, 2016, and 2017 using a crossbow biopsy system as per Hooker, Baird, Al‐Omari, Gowans, and Whitehead (2001). Biopsies were similarly collected from 12 whales in an area off Newfoundland, which was discovered during acoustic surveys of the continental slope in 2015 (L.J. Feyrer, Unpublished Data) and was revisited in 2016–2017. This region has not to our knowledge been previously described as having a significant abundance of northern bottlenose whales, and no bottlenose whales were seen or sampled between the reported areas (Figure 2). Five additional tissue samples were collected during necropsies from northern bottlenose whales that stranded around Atlantic Canada between 1994 and 2005, and were provided by the Newfoundland, Maritimes, and Quebec regions of DFO. Biopsy and stranding samples were preserved in 20% salt‐saturated dimethyl sulphoxide (DMSO) or 70% ethanol and stored at 4°C or −20°C prior to genetic analysis.

### 2.2 DNA extraction

DNA extraction was performed with a glass‐binding/filtration protocol (Elphinstone, Hinten, Anderson, & Nock, 2003) on alcohol or DMSO‐preserved fresh tissues. For archived gum tissue scrapings, DNA extraction was performed using a standard phenol/chloroform protocol (Sambrook, Fritsch, & Maniatis, 1989).

### 2.3 Microsatellite development, and genotyping

Genomic DNA sequences were obtained from two *Hyperoodon ampullatus* collected in 2016, one from Newfoundland and one from the Scotian Shelf. DNAs were extracted using a standard phenol, chloroform, isoamyl‐alcohol technique (Sambrook et al., 1989) from biopsied skin tissue. DNAs were sheared using a Covaris M220 Focussed‐ultrasonicator following the manufacturer's protocols. We used 1 µg input DNA per sample and a target peak of 350 bases in a sample volume of 130 µl (Covaris PN 520045). Covaris settings included 42 s duration, peak power 70, duty factor 20, and cycles per burst 1,000. The sheared library was prepared for sequencing by strict adherence to the Illumina Truseq protocol. The prepared library was enriched via PCR amplification following the Ilumina Truseq protocols and sequenced using an Illumina Miseq Reagent Kit v2 (500‐cycles).

We used MSATCOMMANDER (Faircloth 2008) to identify microsatellites containing sequences and design PCR primers. Search criteria included a minimum of 10 repeats for trinucleotide motifs and a minimum of 20 for dinucleotide repeats with target amplicon lengths between 70 and 130 bp. This amplicon size allows us to sequence the PCR products using Illumina MiSeq Reagent v3 (150‐cycle) kits in a single 150 base read length. While longer reads lengths are possible, the 150‐base read length was cost‐effective, so we designed amplicons with this in mind.

We tested 96 loci: 87 newly developed loci and 8 “legacy” loci previously used by Dalebout et al. (2006), and one sex‐determining locus (CET‐SEX; Konrad, Dupuis, Gero, & Frasier, 2017). Loci were initially vetted versus four samples in three PCRs each containing 32 multiplexed loci per sample using Qiagen Multiplex PCR Kit reagent. Microsatellite alleles were evaluated following allele calling with the software MEGASAT (Zhan et al., 2017, see below). Loci were dropped if they failed to amplify, amplified more than two alleles, or had evidence of null alleles. We retained 58 loci for further investigation. Of the eight legacy loci (see Dalebout et al., 2006), we dropped six for failing the criteria listed above. It is worth noting that “legacy” loci were originally designed for imaging on electrophoretic systems, generally with an amplicon size that exceeds 150 base read length. While it is not always possible to design new priming sites to convert a legacy locus to an NGS locus, it can sometimes be done with additional investment; however, here we considered 58 loci sufficient. We ran these loci versus a larger set of samples (*n* = 153) to further evaluate their performance, dropping loci which proved invariant or amplified unreliably. We retained 49 loci for data collection and analyses (Appendix S1).

### 2.4 Library preparation and allele scoring

Sequencing libraries were prepared using two sequential PCRs. Microsatellite‐specific oligonucleotides were designed with a 5′ tail specific to the Illumina sequencing primers. That is, each left‐microsatellite‐specific oligo was tailed: CCCTACACGACGCTCTTCCG ATCT and each right‐microsatellite‐specific oligo was tailed: GTTCAGACGTGTGCTCTT CCGATCT. These oligos were used in multiplex PCRs to amplify the specific microsatellite loci. Multiplex PCRs were pooled per sample, diluted 10‐fold in water, and used as template for the index PCR. The oligonucleotides in the index‐PCR prime from the tail sequences above, and include a unique 6‐base “index” sequence plus the Illumina adapter sequence which allows the resulting DNA fragment to bind to the flow cell in the sequence step. Thus, the index‐PCR step adds unique index (=barcode) sequences, allowing each individual to be demultiplexed from the sequence output, the only limitation being the number of unique index combinations available and the desired depth of sequencing per sample. Following the index PCR, samples were pooled into libraries (1 μl of PCR product per sample) for sequencing. The resulting libraries were sequenced using Illumina MiSeq Reagent Kit v3 (150‐cycle).

Libraries were loaded with a target depth of 500 reads per sample per locus. We chose this target as a trade‐off between exceeding a minimum depth threshold of 50 reads (per sample per locus) in the weakly amplifying loci versus total sequencing cost per sample. We used Megasat (Zhan et al., 2017) with default allele‐calling parameters, an allowable mismatch of 2 and a minimum depth threshold of 50 reads. MEGASAT output histograms were examined to verify the allele calls, and problem loci were manually edited when necessary.

### 2.5 Mitogenome sequencing and assembly

We prepared a genomic library for each sample by shearing the extracted DNA, attaching sequencing adapters to the DNA fragments, and shotgun sequencing following the methods of Therkildsen and Palumbi (2017). Preliminary sequencing was performed on an Illumina MiSeq at Dalhousie University. Once the sequencing approach was optimized, we conducted additional sequencing using an Illumina HiSeq platform at Genome Québec.

We used a custom pipeline in R (R Core Team, 2018) to isolate mtDNA fragments for each individual and assemble them into complete mitogenomes. We simultaneously trimmed Illumina adapter sequences from all reads and applied stringent thresholds for sequence quality at leading and trailing bases (Q‐score > 25) and over a sliding window of 4 bases (average Q‐score > 23) using Trimmomatic v0.38 (Bolger, Lohse, & Usadel, 2014). We mapped reads from each sample to an *H. ampullatus* reference sequence (GenBank Accession: NC_005273_1) using the Bowtie2 v2.3.4.2 aligner (Langmead & Salzberg, 2012). We then performed an iterative assembly process with MIRA v4.0.2 (Chevreux, Wetter, & Suhai, 1999), first creating a draft guided assembly for each sample using the *H. ampullatus* reference mitogenome and calculating intermediate statistics of assembly performance. Because guided assembly can lead to miscalled insertion and deletion variants, we then performed de novo draft assembly with MIRA for samples that passed an initial completeness threshold of 95%. For samples passing guided assembly but failing de novo assembly, the guided draft assembly was passed to the next step in the pipeline and manually inspected for errors at the end of the assembly process. To account for overhanging genome ends that result from assemblers treating circular mitochondrial genomes as linear, we split draft assemblies at the beginning of the mitochondrial control region and merged these sequences based on their overlap, creating draft mitochondrial genomes of consistent length. To identify errors in each assembly, we remapped reads from each sample to the corresponding assembly with Bowtie2 and used Pilon v1.22 (Walker et al., 2014) to correct miscalled bases, fill gaps, and identify ambiguous bases using read‐based evidence.

### 2.6 Genetic variation

#### 2.6.1 Microsatellites

We calculated indices of genetic variation for each regional group and overall using Hierfstat v0.04.26 (Goudet, 2005), including number of alleles, Simpson's index of allelic diversity, *H*_o_, *H*_e_, and inbreeding coefficients (*F*_IS_). We tested for linkage equilibrium (Agapow & Burt, 2001) among microsatellite loci using Poppr v2.8.0 (Kamvar, Brooks, & Grünwald, 2015). We tested for deviations from Hardy–Weinberg equilibrium (HWE) for each locus by population and overall using Pegas v0.10 (Paradis, 2010).

#### 2.6.2 Relatedness

To assess whether kinship can be evaluated using microsatellite data, we estimated Wang's coefficient of relatedness between all pairs of individuals with 95% confidence intervals and maximum likelihood estimates of inbreeding within populations using Related v1.0 (Pew, Muir, Wang, & Frasier, 2015).

#### 2.6.3 Mitogenomes

After removing technical replicates and duplicates from multiple encounters of the same individuals, we calculated nucleotide diversity, private haplotypes, and Tajima's *D* using Pegas v0.10 (Paradis, 2010) and custom functions for each group and overall. We constructed a phylogenetic tree based on Kimura 2‐parameter distances (Kimura, 1980) using a neighbor‐joining algorithm in Ape v5.0 (Paradis, Claude, & Strimmer, 2004), rooted with the outgroup *Ziphius cavirostris* (Morin, Duchene, Lee, Durban, & Claridge, 2013; GenBank accession: KC776706.1). We evaluated node support by performing 1,000 bootstrap replicates. We inferred an unrooted haplotype network using a median‐joining algorithm (Templeton, Crandall, & Sing, 1992) implemented in Pegas v0.10.

### 2.7 Population structure

#### 2.7.1 Genetic differentiation among stratified samples

To determine whether sampled regions were genetically differentiated while accounting for temporal variation in sampling time, we performed analyses of molecular variance (AMOVAs) with samples stratified by region and year collected using Ade4 v1.7‐13 (Dray & Dufour, 2007). We tested for significant differentiation using 1,000 permutations based on genetic distance from allele frequencies (*F*_ST_) for microsatellites and Kimura 2‐parameter corrected distances (*Φ*_ST_) for mitogenomes (Excoffier, Smouse, & Quattro, 1992).

#### 2.7.2 Bayesian clustering

To infer the number of genetic clusters in northern bottlenose whales and their spatio‐temporal distributions, we performed Bayesian clustering of microsatellite genotypes using Structure v2.3.4 (Falush, Stephens, & Pritchard, 2007; Pritchard, Stephens, Rosenberg, & Donnelly, 2000). We used an admixture model with correlated allele frequencies to allow for mixed ancestry of individuals between genetic clusters. To account for differences in sample sizes and the expectation that both sampling location and year may be informative about ancestry, we used location–year groups as priors (Hubisz, Falush, Stephens, & Pritchard, 2009; Wang, 2017). We averaged model log‐likelihoods and individual assignment coefficients over 10 runs of 100,000 steps following a burn‐in of 100,000 steps for each value of *k* from 1 to 5 and determined the best value of *k* using the ΔK method (Evanno, Regnaut, & Goudet, 2005).

#### 2.7.3 Sex‐biased dispersal

To assess whether sex‐biased dispersal influences population structure, we conducted Bayesian clustering separately for each sex with Structure v2.3.4 and compared distributions of the estimated association between loci (i.e., linkage disequilibrium estimate *r_d_;* Agapow & Burt, 2001) using Poppr v2.8.0 (Kamvar et al., 2015).

#### 2.7.4 Assignment tests

To determine whether individuals recently sampled in Newfoundland, Southern Labrador, the Davis Strait, or from strandings represent migrants from the Northern Labrador or Scotian Shelf, we conducted individual assignment based on reporting groups using a Bayesian approach with Rubias (Anderson & Moran, 2018). We defined Northern Labrador and Scotian Shelf regions as reporting groups based on results of Dalebout et al. (2006) and assessed the accuracy of self‐assignment to these groups as the proportion of correctly assigned individuals using a leave‐one‐out (Anderson, Waples, & Kalinowski, 2008). We explicitly tested whether individuals might not belong to either reporting group, using a Bayesian posterior probability of assignment to 0.70 to minimize the potential for type I error (following Vähä et al., 2011).

### 2.8 Trends in effective population size and demographic reconstruction

We estimated *N*_e_ using the linkage disequilibrium method of Waples and Do (2008), as implemented in NeEstimator V2.1 (Do et al., 2014). To infer the demographic histories and evolutionary trajectories of genetically distinct populations, we used mitogenomes to construct extended Bayesian skyline plots using BEAST v2.4.5 (Bouckaert et al., 2014; Drummond, Suchard, Xie, & Rambaut, 2012). Bayesian skyline analysis assumes panmixia among individuals, and we therefore analyzed the Scotian Shelf separately from all other samples based on clustering analyses from microsatellites and the regional structure identified by AMOVA for mitogenomes. We used a strict molecular clock model of 1.73 × 10^–8^ subs/site/year based on the mean mitochondrial substitution rate in Cetacea (Ho & Lanfear, 2010), with the population model parameter set to 0.5 to account for matrilineal inheritance of mitochondrial DNA. To determine the best evolutionary model for mitogenomes, we first ranked substitution models using bModelTest v0.3.2 (Bouckaert & Drummond, 2017) with a MCMC chain of 10,000,000 states. The best model was HKY with invariable sites (mean proportion = 0.66) and rate heterogeneity (mean shape/rates = 0.2351), which we used with rate and shape estimates as priors for all subsequent analyses. For the extended Bayesian skyline analyses, we ran a chain of 100,000,000 states, sampling every 5,000 states. We assessed convergence in each analysis by comparing posterior distributions in Tracer v1.6 and assessing the effective sample size (ESS > 200) for each estimated parameter.

## 3 RESULTS

### 3.1 DNA extraction and microsatellite validation

DNA obtained from older tissues was of variable quality, and DMSO‐preserved skin tissues tended to yield high‐quality DNA, whereas yields from historical gum samples were poorer, both in terms of DNA quantity and quality.

The average number of microsatellite loci successfully genotyped was 46.4 for northern bottlenose whale samples and 34.8 for other beaked whale species. The success or failure of all microsatellite amplifications and summary statistics such as numbers and sizes of alleles and observed and expected heterozygosities for variable primer pairs that reliably amplified is included in Appendix S1. Of the 58 loci tested, only 37 microsatellite loci were scored unambiguously for the majority of northern bottlenose whale samples and had variation (Table 1; Appendix S1), with <1% missing data per region. The other 21 loci, including legacy loci, were discarded due to poor amplification or lack of variation.

**Table 1:** Genetic diversity of northern bottlenose whale, *Hyperoodon ampullatus*

Region	Mitogenomes	*π*	*N*	*NP*	Haplotype diversity	Microsatellites	Number of alleles	Allelic diversity	Mean *H_o_*	Mean *H_e_*	Private alleles
	*n* (M:F:I)					*n* (M:F:I)					
North Iceland (1967)	5 (3:2)	0.00072	5	1	1.00	7 (3:4)	2.3784	0.3546	0.3430	0.3546	1
Davis Strait (2017–2018)	8 (6:1:1)	0.00114	7	3	0.96	8(6:1:1)	2.5135	0.3688	0.4005	0.3688	1
Northern Labrador (1971)	53 (33:19:1)	0.00096	36	26	0.98	67 (37:29:1)	3.1081	0.3825	0.3800	0.3825	6
Southern Labrador (2003)	3 (2:1)	0.00094	3	1	1.00	3 (2:1)	2.0270	0.3288	0.3784	0.3288	1
Newfoundland (2016–17)	10 (5:5)	0.00103	10	6	1.00	12 (7:5)	2.6486	0.3861	0.3896	0.3861	1
Scotian Shelf (1996–2016)	47 (23:24)	0.00058	15	8	0.87	54 (26:28)	2.8649	0.3837	0.3755	0.3837	4
Stranded (1994–1997)	2 (2:0)	0.00000	1	1	0.00	2 (2:0)	1.9459	0.3547	0.4459	0.3547	0
All	128 (74:52:2)	0.00078	60	NA	0.97	153 (83:68:2)	3.3514	0.3912	0.3796	0.3912	NA

Note

*n* = number of samples, *M* = Male, *F* = Female, I = undetermined sex, *π* = nucleotide diversity, *N* = number of haplotypes, *NP* = number of haplotypes unique to a region. Mean number of alleles (allelic richness), Simpson's allelic diversity, *H_o_* = observed heterozygosity, *H_e_* = expected heterozygosity, alleles unique to each region.

### 3.2 Genetic variation

#### 3.2.1 Microsatellites

Microsatellite diversity at the 37 loci included in population analyses was low, with a maximum of 8 and mean of 3.4 alleles per locus (Appendix S1). Despite this low diversity, the probability of encountering an identical genotype across all 37 loci more than once by chance is 2.68 × 10^–13^, indicating a high power to identify individuals by genotype. We recovered seven genotypes that were sampled twice, representing replicate samples from the same individuals. One of these was a male encountered twice during a single sampling period in 2018 in Davis Strait. The remaining six were within the Scotian Shelf region. Sample metadata and genotypes from the older instance of each resampled individual were excluded from subsequent analyses.

Two microsatellites (Hyam‐108 and Hyam‐114) deviated from Hardy–Weinberg equilibrium across the entire dataset (*p* < .01), and within Scotian Shelf (*p* = .004) and Northern Labrador (*p* = .005) regions, exhibiting homozygote excess. Across all samples, there was no evidence of linkage disequilibrium between pairs of microsatellite loci. When subdivided by region, samples from the Scotian Shelf significantly deviated from independent assortment (*r_d_ *= 0.0114, *p* = .001). This was due to a distribution of *r_d_* with higher than expected values across all pairs of loci rather than strong association between a small number of loci, suggesting that deviation from independent assortment results from demographic processes such as inbreeding, restricted connectivity, or genetic bottlenecks, rather than physical linkage among microsatellite loci (Smith, Smith, O'Rourke, & Spratt, 1993).

The mean of all comparisons for relatedness (${\bar{r}}_{w}$ = −0.0304) was normally distributed and not significantly different from zero, with the mean range of 95% confidence intervals (${\bar{r}}_{w\;-\;\text{hight}}-{\bar{r}}_{w\;-\;\text{low}}$ = 0.7267) spanning values expected for both kin and unrelated pairs. While it is likely that we sampled related pairs of individuals on the Scotian Shelf, the wide distributions of relatedness estimate confidence intervals indicate that these microsatellite data have insufficient power to resolve close kin relationships among individuals.

Levels of microsatellite diversity were similar for each region. For each population and over all data, inbreeding coefficients (*F*_IS_) did not differ significantly from zero, and maximum likelihood estimates of inbreeding did not differ significantly among populations. Simpson's diversity index ranged from 0.35 (North Iceland) to 0.39 (Newfoundland), and observed heterozygosity (*H*_o_) ranged from 0.34 (North Iceland) to 0.40 (Davis Strait). Out of 124 alleles found across all microsatellite loci, only 14 were private or found in only one region. Most of these were found in Northern Labrador (private alleles = 6) or Scotian Shelf (private alleles = 4). All private alleles were rare (mean frequency = 0.0040; maximum frequency = 0.0131).

#### 3.2.2 Mitogenomes

Whole mitochondrial genomes were successfully assembled for 128 individuals, with 110 variable sites over all samples (Feyrer, Bentzen, Whitehead, Patterson, & Einfeldt, 2019). The eight individuals identified as repeat samples using microsatellites were confirmed to have identical mitogenome sequences and were excluded from further analyses. Only one mutation caused a nonsynonymous change in amino acid product, with alternate states coding for tyrosine or cysteine in the coding region for NADH dehydrogenase subunit 6.

**Figure 3:**
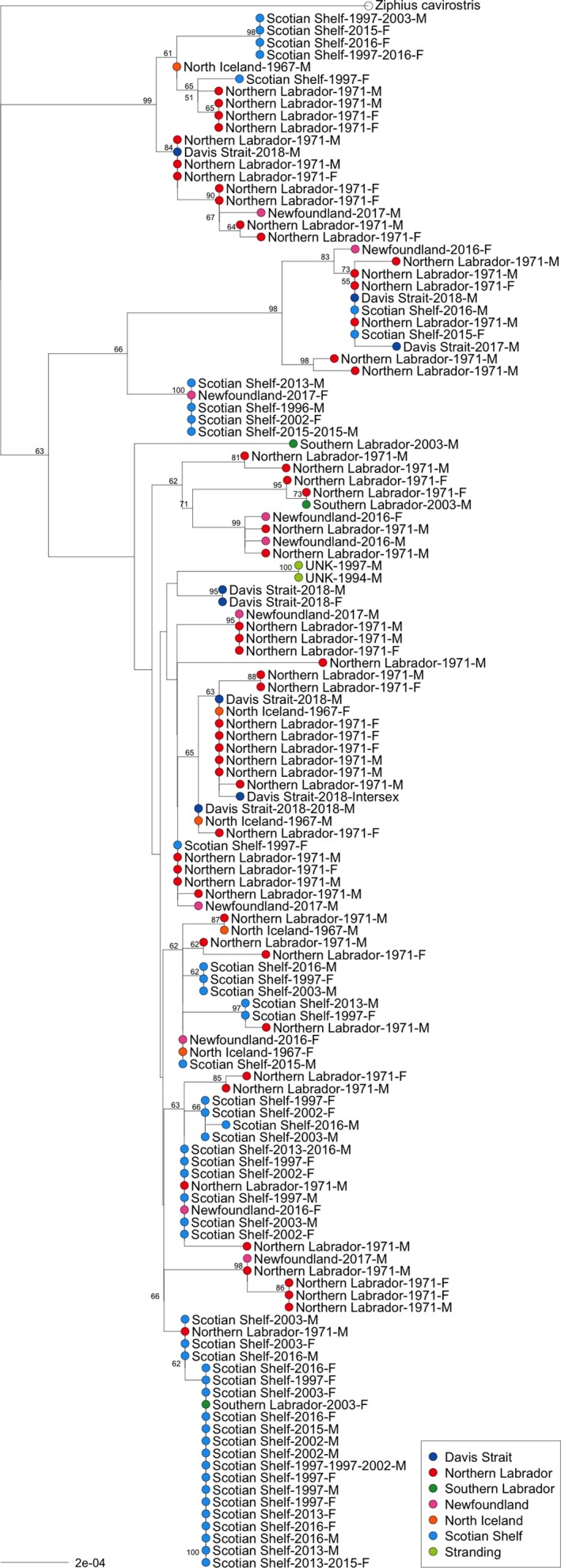
*Hyperoodon ampullatus* mitogenome neighbor‐joining tree from Kimura 2‐parameter distances with bootstrap support (1,000 replicates). Region, year(s) sampled, and sex of each individual specified, with tip color corresponding to region. Red—Northern Labrador, green—Southern Labrador, pink—Newfoundland, orange—Iceland, light blue—Scotian Shelf, dark blue—Davis Strait, and light green—Stranding

**Figure 4:**
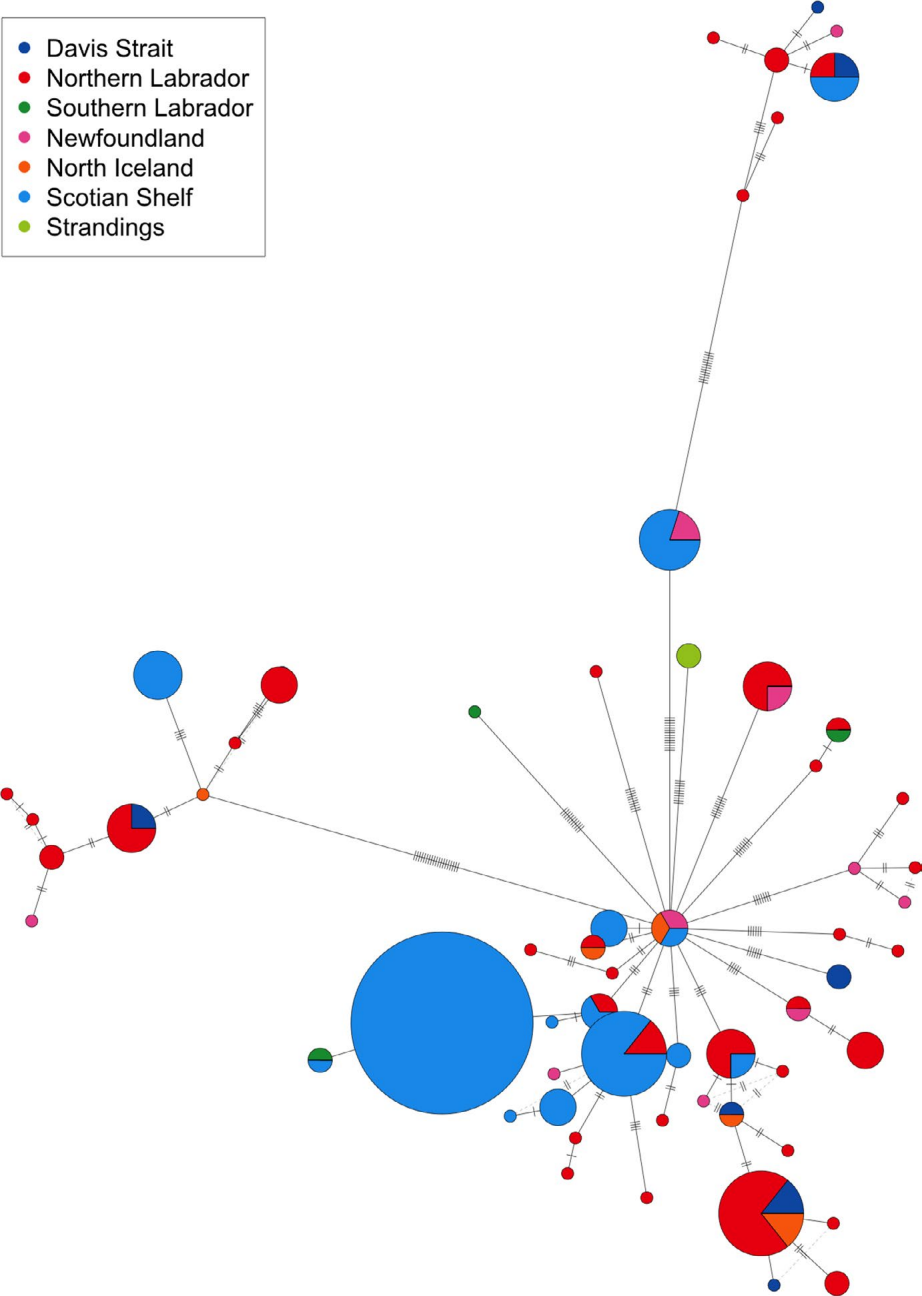
Median‐joining network of *Hyperoodon ampullatus* mitogenome haplotypes. Circles represent haplotypes, colors correspond to sampling region, lines and hash marks depict number of sites differing between haplotypes, and circle area is proportional to number of samples for each haplotype. Red—Northern Labrador, green—Southern Labrador, pink—Newfoundland, orange—Iceland, light blue—Scotian Shelf, dark blue—Davis Strait, and light green—Stranding

Range‐wide mitogenome nucleotide diversity was low (*π* = 0.00078). Regionally, nucleotide diversity was lowest in the Scotian Shelf (*π* = 0.00058), with only 15 unique mitogenome sequences recovered from 47 individuals. Northern Labrador (*n* = 53) had the most haplotypes not found in any other region (*NP* = 26) and had 60% (*N* = 36/60) of the unique mitogenome sequences found in this study. Every mitogenome sequence from Newfoundland was distinct (*n* = 10; *N* = 10), and 60% of these were not found in other regions (*NP* = 6). A bootstrapped phylogenetic tree of mitogenomes resolved several major branches within *H. ampullatus* that were represented in all sampling regions. Out of the 60 mitogenome haplotypes, 14 were found in at least two regions (Figures 3 and 4). Tajima's *D* was only significant when considering all samples (*D *= −1.88; *p* = .034) and was not significant in any individual sampling region.

### 3.3 Population structure

#### 3.3.1 Genetic differentiation among regions

Analyses of molecular variance (AMOVA) revealed low but significant population structure for microsatellites (*F_ST_* = 0.013, *p* = .001). Variation among regions (Φ_region‐total_ = 0.013, *p* = .0010) for microsatellites and for mitogenomes (*Φ*_region‐total_ = 0.055, *p* = .015) was higher than expected at random (Table 2). Pairwise comparisons of differentiation between Scotian Shelf, Northern Labrador, Southern Labrador, Newfoundland, Davis Strait, and Iceland did not identify which regions might drive population structure, with the only significant values of *F_ST_* for microsatellites detected between the two regions with small sample sizes, North Iceland and Southern Labrador (modern) (*F*_ST_ = 0.0707; *p* = .0490) and no significant pairwise *Φ*_ST_ (based on Kimura 2‐parameter distances) for mitogenomes detected in any pairwise comparisons of regions. However, AMOVA of Scotian Shelf against all other individuals grouped together showed significant regional structure for both microsatellites (*Φ*_region‐total_ = 0.018, *p* = .0010) and mitogenome data (*Φ*_region‐total_ = 0.047, *p* = .02).

**Table 2:** AMOVA results for microsatellites and mitogenomes between and within regions for *Hyperoodon ampullatus* in the NW Atlantic

Microsatellites					
~regions (all)	Variance proportion		*Φ*	*HA*	*p*
Between region	**1.2956**	**Phi‐region‐total**	**0.0130**	**Greater**	**.0010**
Between samples within region	2.5027	Phi‐samples‐region	0.0254	Greater	.0609
Within samples	**96.2017**	Phi‐samples‐total	0.0380	Less	**.0190**

~regions (Scotian shelf vs. others)	Variance proportion		*Φ*	*HA*	*p*
Between region	**1.8118**	**Phi‐region‐total**	**0.0181**	**Less**	**.0140**
Between samples within region	2.5273	Phi‐samples‐region	0.0257	Greater	.0619
Within samples	**95.6609**	**Phi‐samples‐total**	**0.0434**	**Greater**	**.0010**

Mitogenomes					
~regions (all)	Variance proportion		*Φ*	*HA*	*p*
Between region	**5.4829**	**Phi‐region‐total**	**0.0548**	**Greater**	**.0150**
Between samples within region	−2.9998	Phi‐samples‐region	−0.0317	Greater	.2957
Within samples	97.5170	Phi‐samples‐total	0.0248	Less	.2468

~regions (Scotian shelf vs. others)	Variance proportion		*Φ*	*HA*	*p*
Between region	**4.7047**	**Phi‐region‐total**	**0.0470**	**Greater**	**.0200**
Between samples within region	−2.6363	Phi‐samples‐region	−0.0277	Greater	.7400
Within samples	97.9315	Phi‐samples‐total	0.0207	Less	.4500

Numbers in bold indicate significance at *p* = 0.05

#### 3.3.2 Bayesian clustering

**Figure 5:**
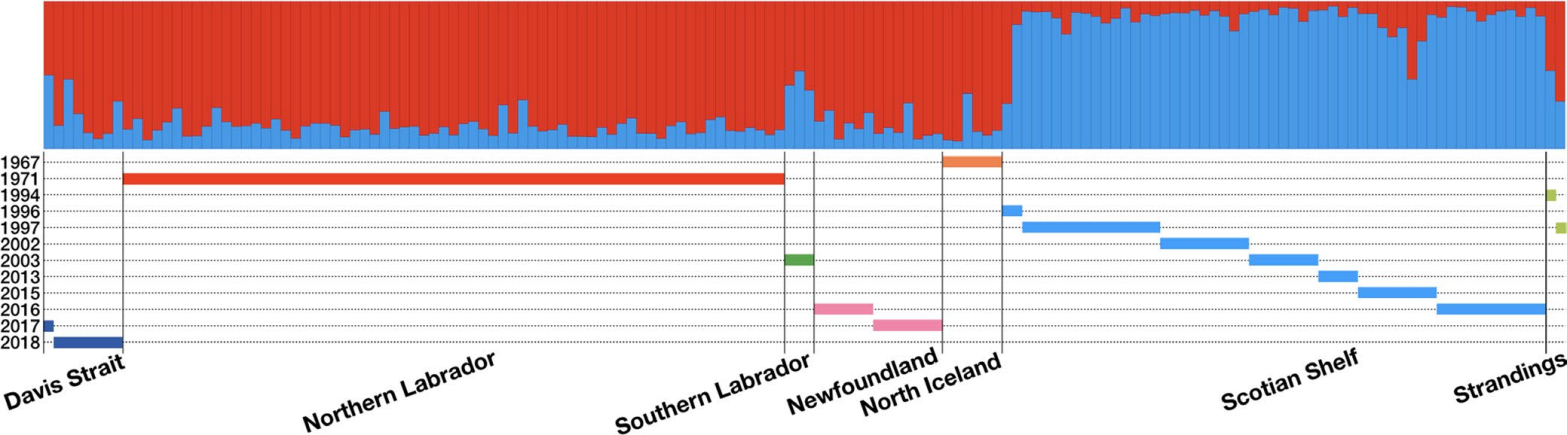
Genetic structure assigned to individual *Hyperoodon ampullatus* using STRUCTURE with location–year group (*n* = 16) priors for *k* = 2. Location of sampling along the *x*‐axis and timeline of sampling below. Red—Northern Labrador, green—Southern Labrador, pink—Newfoundland, orange—Iceland, light blue—Scotian Shelf, dark blue—Davis Strait, and light green—Stranding

To assess population structure while accounting for potential differences between both the locations and years that samples were collected in, we performed Bayesian clustering of microsatellite data in Structure with sampling units defined by location–year. The highest –log Pr(X|*k*) estimates across 10 replicate runs for each value of *k* from 1 to 5 were at *k* = 2, and the ΔK method identified the highest rate of change in –log Pr(X|*k*) estimates for *k* = 2. Assignment probabilities of individuals separated genetic clusters entirely by sample location rather than year (Figure 5), with clustering for *k* = 2 distinguishing individuals from the Scotian Shelf samples from all other individuals. Subsequent runs of only individuals from the Northern region did not detect any finer substructure within our samples. Two individuals from the Scotian Shelf (a female, NBW07‐2015 and a male, HamSH96‐01) had a lower assignment coefficient to the Scotian Shelf cluster (*Q*_1F_ = 0.4728, *Q*_1M_ = 0.3071) than for the other cluster (*Q*_2F_ = 0.5272, *Q*_2M_ = 0.6929), suggesting they may be, or descend from, recent migrants into the Scotian Shelf from another region. Both samples from stranded individuals in the Gulf of St. Lawrence had ambiguous clustering results and could not be reliably assigned to a source location from the STRUCTURE results.

#### 3.3.3 Sex‐biased dispersal

If dispersal is unequal between sexes, the more dispersive sex is expected to have less genetic structure and lower levels of association between loci (i.e., linkage disequilibrium) than the more philopatric sex. We did not find significant differences between the distributions of estimates of linkage disequilibrium among microsatellites in females (*r*_d_ = 0.0039; *p* = .064) or males (*r*_d_ = 0.0017; *p* = .206). STRUCTURE assignment indices identified geographic structure between Scotian Shelf and all other regions in males but not females. This suggests that female northern bottlenose whales may be more dispersive, in contrast to the general male bias pattern of mammals (Mabry, Shelley, Davis, Blumstein, & Vuren, 2013). However, the larger sample size for males (84) than females (70) could influence the ability to detect structure among females. The contrasting results from STRUCTURE and estimates of linkage disequilibrium suggest that the microsatellite data may not have adequate power to assess whether there is sex‐biased dispersal in *H. ampullatus*.

#### 3.3.4 Assignment tests

Self‐assignment of individuals sampled in Scotian Shelf and Northern Labrador had 87.7% accuracy using a leave‐one‐out approach overall, with 85.1% accuracy in Northern Labrador and 90.9% accuracy in Scotian Shelf. Assignment of individuals sampled outside these reporting group areas revealed substantial affinity to Northern Labrador and the potential for intermediate genotypes or presence of unidentified baseline reporting units. Of the three individuals from Southern Labrador, two were assigned to Northern Labrador, and one was not assigned to a reference group. Of the 12 individuals from Newfoundland, seven were assigned to Northern Labrador, one was assigned to Scotian Shelf, and four were not assigned to a reference group. Of the eight individuals from Davis Strait, only two were assigned to Northern Labrador and six were not assigned to a reference group. Of the seven individuals from Iceland, six were assigned to Northern Labrador and one was not assigned to a reference group. Neither of the two strandings were assigned to a reference group.

### 3.4 Effective population size and demographic reconstruction

Estimates of effective population size (*N*_e_) in each region had infinite upper bounds of 95% confidence intervals for all regions except Scotian Shelf (*N*_e_ = 54.8; 95% CI = 43.0–72.7), reflecting limited statistical power to estimate upper bounds. The lower 95% CI bounds for Northern Labrador (*N*_e_ = 495.1; 95% CI = 212.1‐infinite) were higher than the upper bound for Scotian Shelf. Estimates for Newfoundland (*N*_e_ = infinite; 95% CI = 47.8‐infinite), Davis Strait (*N*_e_ = infinite; 95% CI = 24.1‐infinite), and Iceland (*N*_e_ = infinite; 95% CI = 18.4‐infinite) overlapped with estimates for the Scotian Shelf. Due to the low statistical power caused by having only three samples from Southern Labrador, all estimates of *N_e_* for this region were infinite. Estimates for Davis Strait, Northern Labrador, Southern Labrador, and Newfoundland combined (*N*_e_ = 1604.4; 95% CI = 409.5‐infinite) were higher than for each region separately.

**Figure 6:**
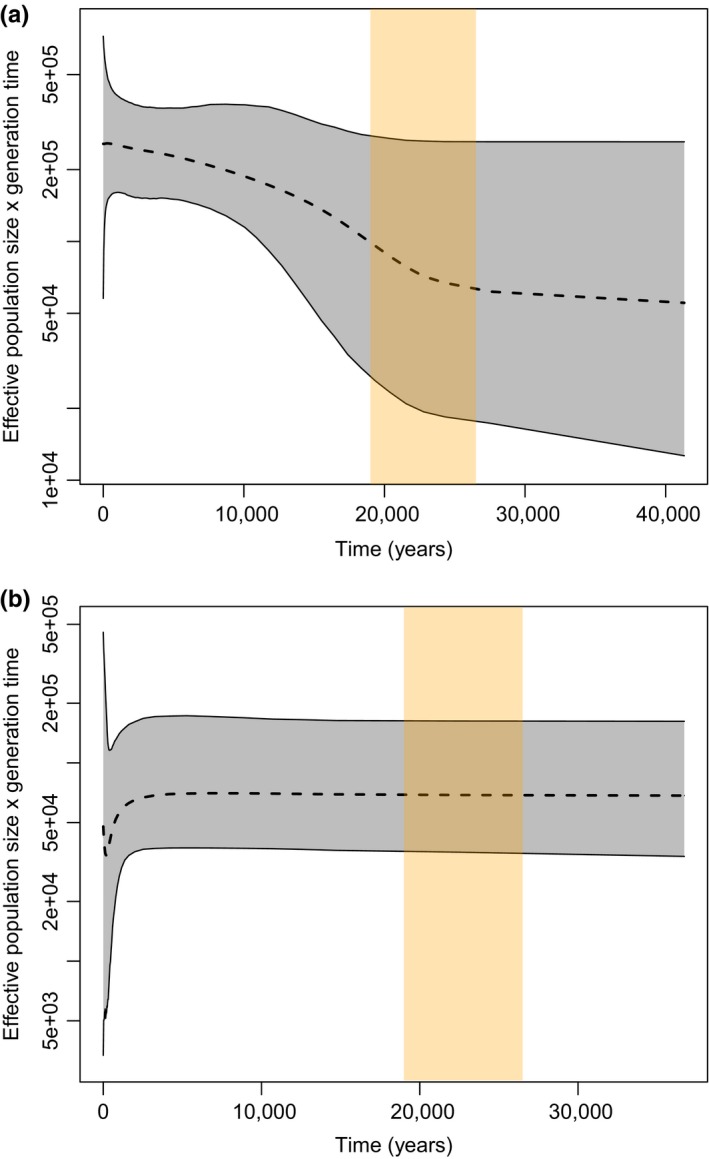
Extended Bayesian skyline plots for *Hyperoodon ampullatus* mitogenomes from (a) Northern region and (b) Scotian Shelf. Dashed line represents median reconstructed Ne, with gray shaded areas representing the 95% (highest posterior density intervals) HPDI. Beige shaded bar indicates the estimated duration of the last glacial maximum. *Y*‐axes are logarithmic. In the Scotian Shelf, effective population size reaches maximum rate of decline at ~350 years and minimum value at ~180 years

Demographic reconstructions differed for the Scotian Shelf population and the group consisting of all other samples (hereafter: Northern region). Because neither group is monophyletic, both are expected to have similar ranges for their time to most recent common ancestor (TMRCA). Consistent with this expectation, the estimated TMRCA for the Northern region was 47.4 kya (95% Highest Posterior Density Interval (HPDI): 32.8–61.5 kya), and the estimated TMRCA for Scotian Shelf was 46.6 kya (95% HPDI: 34.1–61.3 kya). The skyline analysis for the Northern region shows an increasing trend from the estimated time of the last glacial maximum (19.0–26.5 kya) to present times (Figure 6a). In contrast, the extended Bayesian skyline analysis of Scotian Shelf shows a relatively constant population size throughout the last glacial maximum, followed by a sharp decline sometime in the last two centuries (Figure 6b). The maximum rate of decline occurs ~350 years ago, and estimates of effective population size reach a minimum value ~180 years ago. The 95% central posterior density interval reached a minimum range 360 years ago and increased since that time. While median estimates of effective population size increase after the minimum value observed less than 200 years ago, the rapid increase of the 95% central posterior density intervals for years following the minimum accommodate both stable, increasing, and decreasing trends of effective population size. This suggests that whole mitogenomes provide insufficient power to resolve trends in effective population size since the decline.

## 4 DISCUSSION

Understanding the impact of large‐scale removals on species recovery and evolutionary potential ideally involves an assessment of a range of demographic, life history, and genetic correlates (Baker & Clapham, 2004). Because rapid declines in genetic diversity can pose significant risks for small populations, distinguishing between naturally low levels of genetic variation and recent genetic depletion is important. There are several potential causes for low diversity in natural populations, including life history attributes, selective processes, demographic fluctuations, and exploitation bottlenecks. However, for species with long histories of exploitation, such as cetaceans, preharvest population structure or census size is poorly known, and typically few archival specimens are available to reconstruct genetic impacts of harvesting (but see Dufresnes et al., 2018; Phillips et al., 2013). Consequently, studies are increasingly reliant on contemporary sample‐based genetic reconstructions to identify historical bottlenecks, assess genetic resilience and population recovery from past demographic events, and estimate species' evolutionary trajectories (Attard et al., 2015; Carroll et al., 2019; Emami‐Khoyi et al., 2018; Foote et al., 2016). As we have outlined earlier there are a number of reasons that N_e_ and current census size may not be correlated, the uncertainty surrounding estimates of N_e_ and ratios applied to infer true population size (N_c_) is well established (Palstra and Fraser, 2012). Ne cannot be used as a metric to evaluate the impacts of harvesting or the remaining evolutionary potential in a population (Palsbøll, Zachariah Peery, Olsen, Beissinger, & Bérubé, 2013). Methods that consider trends in genetic diversity over time, such as Bayesian skyline and pairwise sequentially Markovian coalescent analyses, provide a more useful historical context for interpreting currently observed patterns. Understanding conservation status should be informed by an appreciation of natural occurring diversity, past demography, and overall trends in population size, which contribute to a species' evolutionary potential and resilience to genetic risks. In the following sections, we evaluate the distinctions between northern bottlenose whale populations, consider the processes that explain their demographic trends, and outline the risks associated with low genetic diversity in light of current conservation concerns for this historically harvested species.

### 4.1 Low genetic diversity

We detected low overall diversity in both microsatellites and mitogenomes of northern bottlenose whales, relative to other species of cetaceans based on a comparative study by Vachon et al. (2018), which accounted for differences in allelic richness between microsatellite loci, sample size, and ascertainment bias. Range‐wide genetic diversity across the full mitogenome is *π* = 0.00078 (*n* = 128), the lowest found for any cetacean; the next lowest is sperm whales (*π* = 0.00096, *n* = 175; Morin et al., 2018). This is consistent with *H. ampullatus* having the lowest known mitochondrial D‐loop nucleotide diversity across 27 species of Cetacea for which this metric is available (Whitehead et al., 2017). The reason for their low diversity is uncertain. Studies of closely related and ecologically similar species of Cuvier's and Gray's beaked whales found higher levels of genetic diversity than *H. ampullatus* and no significant population structure within the same ocean basin, though studies were based on limited mtDNA data (290–590 bp) (Dalebout et al., 2005; Thompson et al., 2016). Both these species of cetaceans have a larger global distribution than *H. ampullatus*, suggesting that geographic distribution across multiple ocean basins may promote genetic diversity, which is supported by other studies that detected a relationship between mtDNA diversity and global latitudinal range (Vachon et al., 2018). Other cetaceans with low genetic diversity and geographic ranges restricted to a single ocean basin include the Narwhal (*Monodon monoceros)* and Commerson's dolphin *(Cephalorhynchus commersonii*Westbury et al., 2019; Whitehead et al., 2017). It is possible that restricted geographic distribution may be correlated with other natural factors, such as low historical population size, evolutionary specializations for prey, or environmental constraints that influence genetic diversity.

### 4.2 Importance of population structure

Concepts for understanding intraspecific population structure can range from demographically independent populations (DIPs), to evolutionarily significant units (ESUs), to subspecies (De Queiroz, 2011; Taylor et al., 2017). Generally, ESUs are groups that are substantially reproductively isolated from other populations, embody an important aspect of the species' evolutionary potential, and may or may not be monophyletic (Moritz, 2002; Palsbøll, Bérubé, & Allendorf, 2007; Taylor et al., 2017). In Canada, national protection for species at risk recognizes “designatable units” (DUs), which are by definition Evolutionarily Significant (COSEWIC, 2018). Dispersal may occur between DUs, so long as it is insufficient to prevent local adaptation (COSEWIC, 2018). The Scotian Shelf population of northern bottlenose whales has been considered a DU in Canada since 2004, due to small population size and isolation from other populations. We detected genetic structure with microsatellite markers and mitogenomes, differentiating the Scotian Shelf population from northern areas and supporting previous work identifying the Scotian Shelf as a separate DU. While not considered here, the importance of population structure and diversity in this species requires full consideration of northern bottlenose whales across the eastern parts of their range.

Previous studies of northern bottlenose whales in the Northwest Atlantic found the Scotian Shelf was genetically distinct from Northern Labrador and Iceland, which was determined by the absence of a single mtDNA haplotype on the Scotian Shelf and significant *F*_ST_ between the Scotian Shelf, Northern Labrador, and Iceland based on microsatellite data (Dalebout et al., 2001, 2006). With additional contemporary samples from all areas (except Iceland), we corroborated the genetic structure between the Scotian Shelf and all other regions through analyses of variation (AMOVA and Bayesian Structure) using 37 microsatellites and the full mitogenome. While all regions shared multiple mitogenome matrilines, we found unique haplotypes in each sampling area. Although excess of a few haplotypes in the Scotian Shelf appears to drive genetic subdivision between regions, due to the numerous haplotypes shared among regions it is unclear how long and to what degree the Scotian Shelf has been isolated. Based on the number of unique haplotypes found in each of the sampling locations across the northern region, it appears all areas contain significant diversity that may be important to the evolutionary potential of the species.

Management concern for the newly discovered whales sampled in the habitat off Newfoundland motivates further consideration of how these individuals fit into currently recognized population structure. The primary genetic distinction detected in our data was between the Scotian Shelf population and all other populations, which collectively formed the Northern region. The few samples from Newfoundland clustered with the Northern region in a Bayesian Structure analysis. However, additional assignment analyses of Newfoundland whales to these two reference groups suggested one grouped with the Scotian Shelf, seven with Northern Labrador, and four were not assigned. These results are not definitive on the population origin of the whales found off Newfoundland, suggesting that it may be an area of mixing between the two currently recognized DUs, and other unknown populations, or possibly represent a newly established population. Although our sample size for Newfoundland is small, due to the high proportion of unique haplotypes in this region and the low overall haplotype variation in this species, Newfoundland appears to represent a source of significant diversity.

Given few barriers in the marine environment, genetic divergence between populations may occur as the result of a number of selective pressures or low population density over evolutionary time. Recent acoustic surveys have documented northern bottlenose whales along the continental slope edge, between the Scotian Shelf and the Northern region (L.J. Feyrer, unpublished data), suggesting that we may not yet have the full picture of contemporary connectivity. Ongoing genetic monitoring is required to resolve whether genetic connectivity is the result of recent historical or contemporary migration or incomplete lineage sorting from a common ancestral population. Previous studies have suggested that genetic separation of the Scotian Shelf from other regions likely predates human exploitation and is not the result of a decline in population size (Dalebout et al., 2006). However, a lack of monophyletic spatial structure with mixed assignment of individuals from sampled regions outside of the Scotian shelf or Northern Labrador suggests there may be ongoing migration among regions. As discussed below, exploitation may have altered the distribution and extent of northern bottlenose whales, and related impacts to their population structure cannot yet be refuted. Given the low overall diversity in northern bottlenose whales, reducing barriers to connectivity between regions—such as those posed by offshore anthropogenic activities—is important for effective conservation with particular concern for the Endangered Scotian Shelf population.

### 4.3 Regional differences in evolutionary trajectories

Few studies of cetaceans have used the full mitogenome to reconstruct demography with Bayesian skyline analyses (but see Cunha et al., 2014; Morin et al., 2013; Morin et al., 2018). Most have relied on comparatively short fragments of mtDNA (414–2494 bp) (Attard et al., 2015; Phillips et al., 2013; Thompson et al., 2016) and were not able to identify more recent impacts within the last 2–10 kya. Demographic reconstructions of other commercially whaled cetaceans, including sperm whales, pygmy blue whales, and bowhead whales, have depicted a gradual signal of population expansion and retraction temporally consistent with historical glacial cycles, but found no substantial evidence of recent declines in diversity that could be attributed to anthropogenic impacts such as whaling (Attard et al., 2015; Morin et al., 2018; Phillips et al., 2013). While the shorter DNA segments used in other studies limited power to distinguish a signal of impacts that may have occurred in the timeframe aligned with human harvesting, here we use >16,000 bp of mtDNA sequenced for 128 individuals to investigate changes in effective population size at temporal scales recent enough to resolve the potential impacts of human activity.

The presence of several lineages in the mitogenome phylogenetic tree (Figure 3), and the star‐shaped expansion signal of the haplotype network (Figure 4), suggests that over their entire range northern bottlenose whale populations have undergone an expansion, likely following colonization of newly available ice‐free habitat after the last glacial maximum (LGM). Consistent with this hypothesis, Tajima's D using all samples was −1.88 (*p* < .05), suggesting either a population expansion or selective sweep occurred. Population expansions following the LGM have been detected in other cetacean species in the North Atlantic (e.g., Sei whales, *Balaenoptera borealis*, Huijser et al., 2018; Minke whales, *Balaenoptera acutorostrata*, Anderwald et al., 2011; white‐sided dolphins, *Lagenorhynchus obliquidens*, Banguera‐Hinestroza, Bjørge, Reid, Jepson, & Hoelzel, 2010).

We conducted separate demographic analyses for the Scotian Shelf and the Northern region due to the assumption of panmixia required for Bayesian skyline analysis. Our reconstruction for the Northern region was consistent with a historical expansion following the LGM, concordant with other studies of cetaceans with temperate ranges overlapping previously glaciated habitats (Figure 6a). The Scotian Shelf population exhibited a more stable effective population size throughout the LGM, followed by a steep decline with a maximum slope occurring ~350 years ago and estimates of effective population size reaching a minimum value within the last 200 years (Figure 6b). The rapid increase in the 95% HPDI after this minimum suggests that inferences of trends in effective population since this decline are unreliable. Although an earlier study by Dalebout et al. (2006) did not find significant evidence of a bottleneck using the M‐ratio and Tajima's *D*, they had significantly lower power in their analyses of 434 bp mtDNA versus the 16,450 bp from the full mitogenome used here. A stable trend across the LGM is plausible, as the Scotian Shelf population exists at the southernmost edge of the species range and may have been least affected by historically colder climate regimes. However, the sudden recent decline in effective population size for the Scotian Shelf population is not consistent with major climatic oscillations. Although the precise timing of the decline within the last 200 years is highly uncertain, human activity is the only major correlate known to have occurred within this period. As whaling removed a large number of whales during this period, we infer that the effects of whaling likely had a greater genetic impact on the more isolated Scotian Shelf population than the Northern population. Below, we further consider the genetic impacts of harvesting and limitations for the recovery of this small population.

### 4.4 Characterizing genetic risk for evaluating species recovery

Genetic risks posed by human harvesting include inbreeding depression and loss of diversity, which can present significant challenges for the recovery of small populations. However, recovery of genetically depauperate species such as cheetah (*Acinonyx jubatus;* Dobrynin et al., 2015) and the elephant seal (*Mirounga angustirostris*; Hoelzel, Fleischer, Campagna, Le Boeuf, & Alvord, 2002) from a few breeding pairs following natural bottlenecks or human‐induced population declines suggests that some species may not have the same genetic load as others, potentially due to low effective population sizes maintained over evolutionary time scales by natural processes (Amos & Balmford, 2001; Amos & Harwood, 1998). Even with naturally low effective population sizes, small peripheral populations are expected to be less resilient to further reductions in genetic diversity due to reduced connectivity and increased potential for inbreeding. Over a period of a few years, commercial whaling for northern bottlenose whales off Nova Scotia removed an equivalent of ~60% of the current Scotian Shelf population size. The whales in the Labrador Sea were also heavily targeted, reducing the closest known potential source of new migrants (O'Brien & Whitehead, 2013; Whitehead & Hooker, 2012). The Bayesian skyline analysis indicates that this level of exploitation coincides with declines in the genetic diversity of the peripheral Scotian Shelf population, suggesting this small population may have an increased risk for inbreeding depression and reduced evolutionary potential to respond to a changing environment.

We were unable to adequately assess the risks associated with inbreeding depression or genetic relatedness in this study due to low overall variation across a panel of 37 microsatellite markers. Different nuclear markers may be able to better distinguish the extent of consanguinity in this small population. However, there are notably fewer mitochondrial haplotypes within the Scotian Shelf population than outside it, suggesting the dominance of a few successful matrilines within this population. Due to the very small population size (*N* ~ 143, O'Brien & Whitehead, 2013) and the low genetic diversity of whales sampled on the Scotian Shelf, there is an increased likelihood of inbreeding in this region relative to others. While we do not have empirical data on whether inbreeding depression is reducing the reproductive output and survival of Scotian Shelf northern bottlenose whales, these factors may have contributed to the slow growth and recovery from whaling observed in this population over the last 50 years (Whitehead & Hooker, 2012).

The Northern region does not appear to have suffered a recent decline in Ne. This may be due to greater connectivity between core areas or inadequate statistical power to detect a recent decline resulting from the large proportion (46%) of whaling era samples in our analysis and low genetic diversity, which is reflected by increasing uncertainty of demographic reconstructions over the last two centuries. Comparing contemporary samples from areas in the Northern region will help validate the lack of a recent bottleneck outside the Scotian Shelf. Stable trends in the effective population size of whales in the Northern region and the new aggregation of whales observed in Newfoundland suggest that in core population centers, the species may be recovering from historical whaling. Comparisons with northern bottlenose whale populations in the eastern North Atlantic may provide additional context for these demographic trends and resolve the phylogeographic history of this species.

## 5 CONCLUSION

Low diversity in *H. ampullatus* is likely naturally occurring, but further population declines or reductions in connectivity could compromise the evolutionary potential of the species and risk the recovery of the more depleted Scotian Shelf population. The genetic risks imposed by harvesting and the slow recovery for the Scotian Shelf population identify a number of considerations that are broadly relevant to the assessment of genetic impacts on commercially exploited species. Distinct populations can respond differently to human exploitation, and determining risk requires an assessment of range‐wide population subdivision and historical trends. We highlight that understanding the evolutionary context and demographic trajectories of distinct populations, using techniques such as Bayesian skyline analysis, can reveal potential genetic risks that can help inform species conservation and management priorities. Population structure may be cryptic and require high‐resolution markers with the power to detect variability, particularly in species with low genetic diversity, which is important to consider when reconstructing historical demography to assess recent human impacts such as exploitation and the recovery of a species across their range.

## CONFLICT OF INTEREST

None declared.

## AUTHOR CONTRIBUTIONS

Laura Joan Feyrer conducted the field work, including biopsy collection from 2015 to 2017, designed the study, and wrote the manuscript. Tony Einfeldt designed and conducted the genetic analyses and contributed to the interpretation of results and writing of the manuscript. Ian Paterson designed the microsatellite primers and conducted most of the laboratory analyses. Paul Bentzen contributed to the analytical design and interpretation of results and revised the manuscript. Hal Whitehead designed the study, contributed to field data collection, and revised the manuscript.

## Data Availability

The data that support the findings of this study have been made openly available online through Dryad (https://doi.org/10.5061/dryad.xgxd254bx), and the pipeline for mtDNA assembly is available on Github at (https://github.com/einfeldt/Hyperoodon). DNA sequences are accessible on Genbank. GenBank accession numbers for mitogenomes are MN536234‐MN536368 and accession numbers for microsatellites are included in Appendix S1.

## References

[ece35813-bib-0001] Agapow, P. , & Burt, A. (2001). Indices of multilocus linkage disequilibrium. Molecular Ecology Notes, 1(1–2), 101–102. 10.1046/j.1471-8278.2000.00014.x

[ece35813-bib-0002] Allendorf, F. W. (2017). Genetics and the conservation of natural populations: Allozymes to genomes. Molecular Ecology, 26(2), 420–430. 10.1111/mec.13948 27933683

[ece35813-bib-0003] Amos, W. , & Balmford, A. (2001). When does conservation genetics matter? Heredity, 87(3), 257–265. 10.1046/j.1365-2540.2001.00940.x 11737272

[ece35813-bib-0004] Amos, W. , & Harwood, J. (1998). Factors affecting levels of genetic diversity in natural populations. Philosophical Transactions of the Royal Society of London. Series B: Biological Sciences, 353(1366), 177–186. 9533122 10.1098/rstb.1998.0200 PMC1692205

[ece35813-bib-0005] Anderson, E. C. , & Moran, B. (2018). rubias: Bayesian inference from the conditional genetic stock identification model. R package version 0.1.0. Retrieved from https://CRAN.R-project.org/package=rubias

[ece35813-bib-0006] Anderson, E. C. , Waples, R. S. , & Kalinowski, S. T. (2008). An improved method for predicting the accuracy of genetic stock identification. Canadian Journal of Fisheries and Aquatic Sciences, 65(7), 1475–1486. 10.1139/F08-049

[ece35813-bib-0007] Anderwald, P. , Daníelsdóttir, A. K. , Haug, T. , Larsen, F. , Lesage, V. , Reid, R. J. , … Hoelzel, A. R. (2011). Possible cryptic stock structure for minke whales in the North Atlantic: Implications for conservation and management. Biological Conservation, 144(10), 2479–2489. 10.1016/j.biocon.2011.07.002

[ece35813-bib-0008] Attard, C. R. M. , Beheregaray, L. B. , Jenner, K. C. S. , Gill, P. C. , Jenner, M.‐N. , Morrice, M. G. , … Möller, L. M. (2015). Low genetic diversity in pygmy blue whales is due to climate‐induced diversification rather than anthropogenic impacts. Biology Letters, 11(5), 20141037 10.1098/rsbl.2014.1037 25948571 PMC4455730

[ece35813-bib-0009] Baker, C. S. , & Clapham, P. J. (2004). Modelling the past and future of whales and whaling. Trends in Ecology and Evolution, 19(7), 365–371. 10.1016/j.tree.2004.05.005 16701287

[ece35813-bib-0010] Banguera‐Hinestroza, E. , Bjørge, A. , Reid, R. J. , Jepson, P. , & Hoelzel, A. R. (2010). The influence of glacial epochs and habitat dependence on the diversity and phylogeography of a coastal dolphin species: *Lagenorhynchus albirostris* . Conservation Genetics, 11(5), 1823–1836. 10.1007/s10592-010-0075-y

[ece35813-bib-0011] Bazin, E. , Glémin, S. , & Galtier, N. (2006). Population size does not influence mitochondrial genetic diversity in animals. Science, 312(5773), 570–572. 10.1126/science.1122033 16645093

[ece35813-bib-0012] Benjaminsen, T. (1972). On the biology of the bottlenose whale, *Hyperoodon ampullatus* (Forster). Norwegian Journal of Zoology, 20, 233–241.

[ece35813-bib-0013] Bolger, A. M. , Lohse, M. , & Usadel, B. (2014). Trimmomatic: A flexible trimmer for Illumina sequence data. Bioinformatics, 30(15), 2114–2120. 10.1093/bioinformatics/btu170 24695404 PMC4103590

[ece35813-bib-0014] Bouckaert, R. R. , & Drummond, A. J. (2017). bModelTest: Bayesian phylogenetic site model averaging and model comparison. BMC Evolutionary Biology, 17(1), 42 10.1186/s12862-017-0890-6 28166715 PMC5294809

[ece35813-bib-0015] Bouckaert, R. , Heled, J. , Kühnert, D. , Vaughan, T. , Wu, C.‐H. , Xie, D. , … Drummond, A. J. (2014). BEAST 2: A software platform for Bayesian evolutionary analysis. PLoS Computational Biology, 10(4), e1003537 10.1371/journal.pcbi.1003537 24722319 PMC3985171

[ece35813-bib-0016] Bürger, R. , & Lynch, M. (1995). Evolution and extinction in a changing environment: A quantitative‐genetic analysis. Evolution, 49(1), 151–163. 28593664 10.1111/j.1558-5646.1995.tb05967.x

[ece35813-bib-0017] Carroll, E. L. , Alderman, R. , Bannister, J. L. , Bérubé, M. , Best, P. B. , Boren, L. , … Gaggiotti, O. E. (2019). Incorporating non‐equilibrium dynamics into demographic history inferences of a migratory marine species. Heredity, 122(1), 53–68. 10.1038/s41437-018-0077-y 29720718 PMC6288115

[ece35813-bib-0018] Chevreux, B. , Wetter, T. , & Suhai, S. (1999). Genome sequence assembly using trace signals and additional sequence information. Computer Science and Biology: Proceedings of the German Conference on Bioinformatics (GCB), 99(1), 45–56.

[ece35813-bib-0019] Christensen, I. (1973). Age determination, age distribution and growth of bottlenose whales, *Hyperoodon ampullatus* (Forster), in the Labrador Sea. Norwegian Journal of Zoology, 21, 331–340.

[ece35813-bib-0020] Christensen, I. (1975). Preliminary report on the Norwegian fishery for small whales: Expansion of Norwegian whaling to arctic and northwest Atlantic waters, and Norwegian investigations of the biology of small whales. Journal of the Fisheries Board of Canada, 32(7), 1083–1094. 10.1139/f75-129

[ece35813-bib-0021] COSEWIC (2011). COSEWIC assessment and status report on the Northern Bottlenose Whale *Hyperoodon ampullatus* in Canada. Ottawa, ON: Committee on the Status of Endangered Wildlife in Canada.

[ece35813-bib-0022] COSEWIC . (2018, December 28). COSEWIC guidelines for recognizing designatable units approved November 2015 [Internet document]. Retrieved from http://www.canada.ca/en/environment-climate-change/services/committee-status-endangered-wildlifeguidelines-recognizing-designatable-units.html

[ece35813-bib-0023] Cunha, H. A. , Medeiros, B. V. , Barbosa, L. A. , Cremer, M. J. , Marigo, J. , Lailson‐Brito, J. , … Solé‐Cava, A. M. (2014). Population structure of the endangered franciscana dolphin (*Pontoporia blainvillei*): Reassessing management units. PLoS ONE, 9(1), e85633 10.1371/journal.pone.0085633 24497928 PMC3908959

[ece35813-bib-0024] Dalebout, M. K. , Hooker, S. K. , & Christensen, I. (2001). Genetic diversity and population structure among northern bottlenose whales, *Hyperoodon ampullatus*, in the western North Atlantic Ocean. Canadian Journal of Zoology, 79(3), 478–484.

[ece35813-bib-0025] Dalebout, M. L. , Robertson, K. M. , Frantzis, A. , Engelhaupt, D. A. N. , Mignucci‐Giannoni, A. A. , Rosario‐Delestre, R. J. , & Baker, C. S. (2005). Worldwide structure of mtDNA diversity among Cuvier's beaked whales (*Ziphius cavirostris*): Implications for threatened populations. Molecular Ecology, 14(11), 3353–3371. 10.1111/j.1365-294X.2005.02676.x 16156808

[ece35813-bib-0026] Dalebout, M. L. , Ruzzante, D. E. , Whitehead, H. , & Øien, N. I. (2006). Nuclear and mitochondrial markers reveal distinctiveness of a small population of bottlenose whales (*Hyperoodon ampullatus*) in the western North Atlantic. Molecular Ecology, 15(11), 3115–3129. 10.1111/j.1365-294X.2006.03004.x 16968258

[ece35813-bib-0027] Dalebout, M. L. , Steel, D. , & Baker, C. S. (2008). Phylogeny of the beaked whale genus Mesoplodon (Ziphiidae: Cetacea) revealed by nuclear introns: Implications for the evolution of male tusks. Systematic Biology, 57(6), 857–875. 10.1080/10635150802559257 19085329

[ece35813-bib-0028] De Bruyn, M. , Hall, B. L. , Chauke, L. F. , Baroni, C. , Koch, P. L. , & Hoelzel, A. R. (2009). Rapid response of a marine mammal species to Holocene climate and habitat change. PLoS Genetics, 5(7), e1000554 10.1371/journal.pgen.1000554 19593366 PMC2700269

[ece35813-bib-0029] De Queiroz, K. (2011). Branches in the lines of descent: Charles Darwin and the evolution of the species concept. Biological Journal of the Linnean Society, 103, 19–35. 10.1111/j.1095-8312.2011.01634.x

[ece35813-bib-0030] Do, C. , Waples, R. S. , Peel, D. , Macbeth, G. M. , Tillett, B. J. , & Ovenden, J. R. (2014). NeEstimator V2: Re‐implementation of software for the estimation of contemporary effective population size (Ne) from genetic data. Molecular Ecology Resources, 14, 209–214. 23992227 10.1111/1755-0998.12157

[ece35813-bib-0031] Dobrynin, P. , Liu, S. , Tamazian, G. , Xiong, Z. , Yurchenko, A. A. , Krasheninnikova, K. , … O'Brien, S. J. (2015). Genomic legacy of the African cheetah, *Acinonyx * *jubatus* . Genome Biology, 16(1), 277 10.1186/s13059-015-0837-4 26653294 PMC4676127

[ece35813-bib-0032] Dray, S. , & Dufour, A. B. (2007). The ade4 package: Implementing the duality diagram for ecologists. Journal of Statistical Software, 22(4), 1–20.

[ece35813-bib-0033] Drummond, A. J. , Suchard, M. A. , Xie, D. , & Rambaut, A. (2012). Bayesian phylogenetics with BEAUti and the BEAST 1.7. Molecular Biology and Evolution, 29(8), 1969–1973. 10.1093/molbev/mss075 22367748 PMC3408070

[ece35813-bib-0034] Dufresnes, C. , Miquel, C. , Remollino, N. , Biollaz, F. , Salamin, N. , Taberlet, P. , & Fumagalli, L. (2018). Howling from the past: Historical phylogeography and diversity losses in European grey wolves. Proceedings of the Royal Society B: Biological Sciences, 285(1884), 20181148–20181210. 10.1098/rspb.2018.1148 PMC6111155 30068681

[ece35813-bib-0035] Ellegren, H. , & Galtier, N. (2016). Determinants of genetic diversity. Nature Reviews Genetics, 17(7), 422–433. 10.1038/nrg.2016.58 27265362

[ece35813-bib-0036] Elphinstone, M. S. , Hinten, G. N. , Anderson, M. J. , & Nock, C. J. (2003). An inexpensive and high‐throughput procedure to extract and purify total genomic DNA for population studies. Molecular Ecology Notes, 3(2), 317–320. 10.1046/j.1471-8286.2003.00397.x

[ece35813-bib-0037] Emami‐Khoyi, A. , Paterson, A. M. , Hartley, D. A. , Boren, L. J. , Cruickshank, R. H. , Ross, J. G. , … Else, T. A. (2018). Mitogenomics data reveal effective population size, historical bottlenecks, and the effects of hunting on New Zealand fur seals (*Arctocephalus forsteri*). Mitochondrial DNA Part A, 29(4), 567–580. 10.1080/24701394.2017.1325478 28539070

[ece35813-bib-0038] Evanno, G. , Regnaut, S. , & Goudet, J. (2005). Detecting the number of clusters of individuals using the software STRUCTURE: A simulation study. Molecular Ecology, 14(8), 2611–2620. 10.1111/j.1365-294X.2005.02553.x 15969739

[ece35813-bib-0039] Excoffier, L. , Smouse, P. E. , & Quattro, J. M. (1992). Analysis of molecular variance inferred from metric distances among DNA haplotypes: Application to human mitochondrial DNA restriction data. Genetics, 131, 479–491. 1644282 10.1093/genetics/131.2.479 PMC1205020

[ece35813-bib-0040] Falush, D. , Stephens, M. , & Pritchard, J. K. (2007). Inference of population structure using multilocus genotype data: Dominant markers and null alleles. Molecular Ecology Notes, 7(4), 574–578. 10.1111/j.1471-8286.2007.01758.x 18784791 PMC1974779

[ece35813-bib-0041] Feyrer, L. J. , Bentzen, P. , Whitehead, H. , Patterson, I. G. , & Einfeldt, A. (2019). Data from: Evolutionary impacts differ between two exploited populations of northern bottlenose whale (*Hyperoodon ampullatus*). GenBank accessions for mitogenomes: MN536234‐MN536368. 10.1002/ece3.5813 PMC6912904 31871667

[ece35813-bib-0042] Foote, A. D. , Vijay, N. , Ávila‐Arcos, M. C. , Baird, R. W. , Durban, J. W. , Fumagalli, M. , … Wolf, J. B. W. (2016). Genome‐culture coevolution promotes rapid divergence of killer whale ecotypes. Nature Communications, 7, 11693 10.1038/ncomms11693 PMC4895049 27243207

[ece35813-bib-0043] Frankham, R. (2005). Genetics and extinction. Biological Conservation, 126(2), 131–140. 10.1016/j.biocon.2005.05.002

[ece35813-bib-0044] Frankham, R. (2015). Genetic rescue of small inbred populations: Meta‐analysis reveals large and consistent benefits of gene flow. Molecular Ecology, 24(11), 2610–2618. 10.1111/mec.13139 25740414

[ece35813-bib-0045] Gol'din, P. (2014). “Antlers inside”: Are the skull structures of beaked whales (Cetacea: Ziphiidae) used for echoic imaging and visual display? Biological Journal of the Linnean Society, 113(2), 510–515.

[ece35813-bib-0046] Goudet, J. (2005). Hierfstat, a package for R to compute and test variance components and F‐statistics. Molecular Ecology Notes, 5, 184–186.

[ece35813-bib-0047] Gowans, S. , Whitehead, H. , & Hooker, S. K. (2001). Social organization in northern bottlenose whales, *Hyperoodon ampullatus*: Not driven by deep‐water foraging? Animal Behaviour, 62(2), 369–377. 10.1006/anbe.2001.1756

[ece35813-bib-0048] Hewitt, G. (2000). The genetic legacy of the Quaternary ice ages. Nature, 405(6789), 907 10.1038/35016000 10879524

[ece35813-bib-0049] Ho, S. Y. , & Lanfear, R. (2010). Improved characterisation of among‐lineage rate variation in cetacean mitogenomes using codon‐partitioned relaxed clocks. Mitochondrial DNA, 21(3–4), 138–146. 10.3109/19401736.2010.494727 20795783

[ece35813-bib-0050] Hoelzel, A. R. , Fleischer, R. C. , Campagna, C. , Le Boeuf, B. J. , & Alvord, G. (2002). Impact of a population bottleneck on symmetry and genetic diversity in the northern elephant seal. Journal of Evolutionary Biology, 15(4), 567–575. 10.1046/j.1420-9101.2002.00419.x

[ece35813-bib-0051] Hooker, S. K. , Baird, R. W. , Al‐Omari, S. , Gowans, S. , & Whitehead, H. (2001). Behavioral reactions of northern bottlenose whales (*Hyperoodon ampullatus*) to biopsy darting and tag attachment procedures. Fishery Bulletin, 99(2), 303–308.

[ece35813-bib-0052] Hooker, S. K. , Iverson, S. J. , Ostrom, P. , & Smith, S. C. (2001). Diet of northern bottlenose whales inferred from fatty‐acid and stable‐isotope analyses of biopsy samples. Canadian Journal of Zoology, 79(8), 1442–1454. 10.1139/z01-096

[ece35813-bib-0053] Hubisz, M. J. , Falush, D. , Stephens, M. , & Pritchard, J. K. (2009). Inferring weak population structure with the assistance of sample group information. Molecular Ecology Resources, 9(5), 1322–1332. 10.1111/j.1755-0998.2009.02591.x 21564903 PMC3518025

[ece35813-bib-0054] Huijser, L. A. E. , Bérubé, M. , Cabrera, A. A. , Prieto, R. , Silva, M. A. , Robbins, J. , … Palsbøll, P. J. (2018). Population structure of North Atlantic and North Pacific sei whales (*Balaenoptera borealis*) inferred from mitochondrial control region DNA sequences and microsatellite genotypes. Conservation Genetics, 19(4), 1007–1024. 10.1007/s10592-018-1076-5

[ece35813-bib-0055] Hutchings, J. A. , Butchart, S. H. M. , Collen, B. , Schwartz, M. K. , & Waples, R. S. (2012). Red flags: Correlates of impaired species recovery. Trends in Ecology and Evolution, 27(10), 542–546. 10.1016/j.tree.2012.06.005 22784411

[ece35813-bib-0056] IUCN (2018). 2018 IUCN red list of threatened species. Retrieved from http://www.iucnredlist.org

[ece35813-bib-0057] Jenkins, T. L. , Castilho, R. , & Stevens, J. R. (2018). Meta‐analysis of northeast Atlantic marine taxa shows contrasting phylogeographic patterns following post‐LGM expansions. PeerJ, 6, e5684 10.7717/peerj.5684 30280047 PMC6166638

[ece35813-bib-0058] Kamvar, Z. N. , Brooks, J. C. , & Grünwald, N. J. (2015). Novel R tools for analysis of genome‐wide population genetic data with emphasis on clonality. Frontiers in Genetics, 6, 208 10.3389/fgene.2015.00208 26113860 PMC4462096

[ece35813-bib-0059] Keller, L. F. , & Waller, D. M. (2002). Inbreeding effects in wild populations. Trends in Ecology and Evolution, 17(5), 230–241. 10.1016/S0169-5347(02)02489-8

[ece35813-bib-0060] Kimura, M. A. (1980). A simple method for estimating evolutionary rates of base substitutions through comparative studies of nucleotide sequences. Journal of Molecular Evolution, 16(2), 111–120. 7463489 10.1007/BF01731581

[ece35813-bib-0061] Konrad, C. M. , Dupuis, A. , Gero, S. , & Frasier, T. (2017). A sexing technique for highly degraded cetacean DNA. Aquatic Mammals, 43(6), 655–660. 10.1578/AM.43.6.2017.655

[ece35813-bib-0062] Lacy, R. C. (1997). Importance of genetic variation to the viability of mammalian populations. Journal of Mammalogy, 78(2), 320–335. 10.2307/1382885

[ece35813-bib-0063] Langmead, B. , & Salzberg, S. L. (2012). Fast gapped‐read alignment with Bowtie 2. Nature Methods, 9(4), 357 10.1038/nmeth.1923 22388286 PMC3322381

[ece35813-bib-0064] Leffler, E. M. , Bullaughey, K. , Matute, D. R. , Meyer, W. K. , Segurel, L. , Venkat, A. , … Przeworski, M. (2012). Revisiting an old riddle: What determines genetic diversity levels within species? PLoS Biology, 10(9), e1001388. 22984349 10.1371/journal.pbio.1001388 PMC3439417

[ece35813-bib-0065] Mabry, K. E. , Shelley, E. L. , Davis, K. E. , Blumstein, D. T. , & Van Vuren, D. H. (2013). Social mating system and sex‐biased dispersal in mammals and birds: A phylogenetic analysis. PLoS ONE, 8(3), e57980 10.1371/journal.pone.0057980 23483957 PMC3590276

[ece35813-bib-0066] Martien, K. K. , Chivers, S. J. , Baird, R. W. , Archer, F. I. , Gorgone, A. M. , Hancock‐Hanser, B. L. , … Taylor, B. L. (2014). Nuclear and mitochondrial patterns of population structure in North Pacific false killer whales (*Pseudorca crassidens*). Journal of Heredity, 105(5), 611–626. 10.1093/jhered/esu029 24831238

[ece35813-bib-0067] Mitchell, E. (1977). Evidence that the northern bottlenose whale is depleted. Report of the International Whaling Commission, 27, 195–203.

[ece35813-bib-0068] Morin, P. A. , Duchene, S. , Lee, N. , Durban, J. , & Claridge, D. (2013). Preliminary analysis of mitochondrial genome phylogeography of Blainvilles', Cuvier's and Gervais' beaked whales. Report to the Scientific Committee of the International Whaling Commission (No. SC/64/SM14), (p. 17).

[ece35813-bib-0069] Morin, P. A. , Foote, A. D. , Baker, C. S. , Hancock‐Hanser, B. L. , Kaschner, K. , Mate, B. R. , … Alexander, A. (2018). Demography or selection on linked cultural traits or genes? Investigating the driver of low mtDNA diversity in the sperm whale using complementary mitochondrial and nuclear genome analyses. Molecular Ecology, 27(11), 2604–2619. 10.1111/mec.14698 29675902

[ece35813-bib-0070] Moritz, C. (2002). Strategies to protect biological diversity and the evolutionary processes that sustain it. Systematic Biology, 51(2), 238–254. 10.1080/10635150252899752 12028731

[ece35813-bib-0071] Moura, A. E. , Janse van Rensburg, C. , Pilot, M. , Tehrani, A. , Best, P. B. , Thornton, M. , … Hoelzel, A. R. (2014). Killer whale nuclear genome and mtDNA reveal widespread population bottleneck during the last glacial maximum. Molecular Biology and Evolution, 31(5), 1121–1131. 10.1093/molbev/msu058 24497033 PMC3995335

[ece35813-bib-0072] O'Brien, K. , & Whitehead, H. (2013). Population analysis of Endangered northern bottlenose whales on the Scotian Shelf seven years after the establishment of a Marine Protected Area. Endangered Species Research, 21(3), 273–284. 10.3354/esr00533

[ece35813-bib-0073] O'Grady, J. J. , Brook, B. W. , Reed, D. H. , Ballou, J. D. , Tonkyn, D. W. , & Frankham, R. (2006). Realistic levels of inbreeding depression strongly affect extinction risk in wild populations. Biological Conservation, 133(1), 42–51. 10.1016/j.biocon.2006.05.016

[ece35813-bib-0074] Palsbøll, P. , Bérubé, M. , & Allendorf, F. (2007). Identification of management units using population genetic data. Trends in Ecology and Evolution, 22(1), 11–16. 10.1016/j.tree.2006.09.003 16982114

[ece35813-bib-0075] Palsbøll, P. J. , Zachariah Peery, M. , Olsen, M. T. , Beissinger, S. R. , & Bérubé, M. (2013). Inferring recent historic abundance from current genetic diversity. Molecular Ecology, 22(1), 22–40. 10.1111/mec.12094 23181682

[ece35813-bib-0076] Palstra, F. P. , & Fraser, D. J. (2012). Effective/census population size ratio estimation: A compendium and appraisal. Ecology and Evolution, 2(9), 2357–2365. 10.1002/ece3.329 23139893 PMC3488685

[ece35813-bib-0077] Paradis, E. (2010). pegas: An R package for population genetics with an integrated–modular approach. Bioinformatics, 26(3), 419–420. 10.1093/bioinformatics/btp696 20080509

[ece35813-bib-0078] Paradis, E. , Claude, J. , & Strimmer, K. (2004). APE: Analyses of phylogenetics and evolution in R language. Bioinformatics, 20(2), 289–290. 10.1093/bioinformatics/btg412 14734327

[ece35813-bib-0079] Paul, A. , & Schäfer‐Neth, C. (2003). Modeling the water masses of the Atlantic Ocean at the Last Glacial Maximum. Paleoceanography, 18(3), 1–16. 10.1029/2002PA000783

[ece35813-bib-0080] Pew, J. , Muir, P. H. , Wang, J. , & Frasier, T. R. (2015). related: An R package for analysing pairwise relatedness from codominant molecular markers. Molecular Ecology Resources, 15(3), 557–561. 10.1111/1755-0998.12323 25186958

[ece35813-bib-0081] Phillips, C. D. , Hoffman, J. I. , George, J. C. , Suydam, R. S. , Huebinger, R. M. , Patton, J. C. , & Bickham, J. W. (2013). Molecular insights into the historic demography of bowhead whales: Understanding the evolutionary basis of contemporary management practices. Ecology and Evolution, 3(1), 18–37. 10.1002/ece3.374 PMC3568839 23403722

[ece35813-bib-0082] Pritchard, J. K. , Stephens, M. , Rosenberg, N. A. , & Donnelly, P. (2000). Association mapping in structured populations. The American Journal of Human Genetics, 67(1), 170–181. 10.1086/302959 10827107 PMC1287075

[ece35813-bib-0083] R Core Team (2018). R: A language and environment for statistical computing. Vienna, Austria: R Foundation for Statistical Computing Retrieved from http://www.R-project.org/

[ece35813-bib-0084] Reeves, R. R. , Mitchell, E. , & Whitehead, H. (1993). Status of Northern Bottlenose whale, *Hyperoodon * *ampullatus* . Canadian Field Naturalist, 107(4), 490–508.

[ece35813-bib-0085] Romiguier, J. , Gayral, P. , Ballenghien, M. , Bernard, A. , Cahais, V. , Chenuil, A. , … Galtier, N. (2014). Comparative population genomics in animals uncovers the determinants of genetic diversity. Nature, 515(7526), 261 10.1038/nature13685 25141177

[ece35813-bib-0086] Sambrook, J. , Fritsch, E. F. , & Maniatis, T. (1989). Molecular Cloning: A laboratory manual. New York, NY: Cold Spring Harbor Laboratory.

[ece35813-bib-0087] Smith, J. M. , Smith, N. H. , O'Rourke, M. , & Spratt, B. G. (1993). How clonal are bacteria? Proceedings of the National Academy of Sciences of the United States of America, 90(10), 4384–4388. 10.1073/pnas.90.10.4384 8506277 PMC46515

[ece35813-bib-0088] Taylor, B. L. , Chivers, S. J. , Larese, J. , & Perrin, W. F. (2007). Generation length and percent mature estimates for IUCN assessments of cetaceans. NOAA, NMFS, Southwest Fisheries Science Center Administrative Report, LJ‐07–01, 21.

[ece35813-bib-0089] Taylor, B. L. , Perrin, W. F. , Reeves, R. R. , Rosel, P. E. , Wang, J. Y. , Cipriano, F. , … Brownell, R. L. (2017). Why we should develop guidelines and quantitative standards for using genetic data to delimit subspecies for data‐poor organisms like cetaceans. Marine Mammal Science, 33(S1), 12–26. 10.1111/mms.12413

[ece35813-bib-0090] Templeton, A. R. , Crandall, K. A. , & Sing, C. F. (1992). A cladistic analysis of phenotypic association with haplotypes inferred from restriction endonuclease mapping and DNA sequence data. III. Cladogram estimation. Genetics, 132, 619–635. 1385266 10.1093/genetics/132.2.619 PMC1205162

[ece35813-bib-0091] Therkildsen, N. O. , & Palumbi, S. R. (2017). Practical low‐coverage genomewide sequencing of hundreds of individually barcoded samples for population and evolutionary genomics in nonmodel species. Molecular Ecology Resources, 17(2), 194–208. 10.1111/1755-0998.12593 27496322

[ece35813-bib-0092] Thompson, K. F. , Patel, S. , Baker, C. S. , Constantine, R. , & Millar, C. D. (2016). Bucking the trend: Genetic analysis reveals high diversity, large population size and low differentiation in a deep ocean cetacean. Heredity, 116(3), 277–285. 10.1038/hdy.2015.99 26626574 PMC4806577

[ece35813-bib-0093] Vachon, F. , Whitehead, H. , & Frasier, T. R. (2018). What factors shape genetic diversity in cetaceans? Ecology and Evolution, 8(3), 1554–1572. 10.1002/ece3.3727 29435232 PMC5792597

[ece35813-bib-0094] Vähä, J. P. , Erkinaro, J. , Niemelä, E. , Primmer, C. R. , Saloniemi, I. , Johansen, M. , … Brørs, S. (2011). Temporally stable population‐specific differences in run timing of one‐sea‐winter Atlantic salmon returning to a large river system. Evolutionary Applications, 4(1), 39–53. 10.1111/j.1752-4571.2010.00131.x 25567952 PMC3352515

[ece35813-bib-0095] Walker, B. J. , Abeel, T. , Shea, T. , Priest, M. , Abouelliel, A. , Sakthikumar, S. , … Earl, A. M. (2014). Pilon: An integrated tool for comprehensive microbial variant detection and genome assembly improvement. PLoS ONE, 9(11), e112963 10.1371/journal.pone.0112963 25409509 PMC4237348

[ece35813-bib-0096] Wang, J. (2017). The computer program STRUCTURE for assigning individuals to populations: Easy to use but easier to misuse. Molecular Ecology Resources, 17(5), 981–990. 28028941 10.1111/1755-0998.12650

[ece35813-bib-0097] Waples, R. S. , & Do, C. H. I. (2008). LDNE: A program for estimating effective population size from data on linkage disequilibrium. Molecular Ecology Resources, 8(4), 753–756. 21585883 10.1111/j.1755-0998.2007.02061.x

[ece35813-bib-0098] Westbury, M. V. , Petersen, B. , Garde, E. , Heide‐Jørgensen, M. P. , & Lorenzen, E. D. (2019). Narwhal genome reveals long‐term low genetic diversity despite current large abundance size. iScience, 15, 592–599. 10.1016/j.isci.2019.03.023 31054839 PMC6546971

[ece35813-bib-0099] Whitehead, H. (1998). Cultural selection and genetic diversity in matrilineal whales. Science, 282(5394), 1708–1711. 10.1126/science.282.5394.1708 9831562

[ece35813-bib-0100] Whitehead, H. , Faucher, A. , Gowans, S. , & McCarrey, S. (1997). Status of the Northern Bottlenose Whale, *Hyperoodon ampullatus*, in the Gully, Nova Scotia. Canadian Field Naturalist, 111(2), 287–292.

[ece35813-bib-0101] Whitehead, H. , & Hooker, S. K. (2012). Uncertain status of the northern bottlenose whale Hyperoodon ampullatus: Population fragmentation, legacy of whaling and current threats. Endangered Species Research, 19(1), 47–61. 10.3354/esr00458

[ece35813-bib-0102] Whitehead, H. , Vachon, F. , & Frasier, T. R. (2017). Cultural hitchhiking in the matrilineal whales. Behavior Genetics, 47(3), 324–334. 10.1007/s10519-017-9840-8 28275880

[ece35813-bib-0103] Willi, Y. , Van Buskirk, J. , & Hoffmann, A. A. (2006). Limits to the adaptive potential of small populations. Annual Review of Ecology, Evolution, and Systematics, 37(1), 433–458. 10.1146/annurev.ecolsys.37.091305.110145

[ece35813-bib-0104] Wilson Sayres, M. A. (2018). Genetic diversity on the sex chromosomes. Genome Biology and Evolution, 10(4), 1064–1078. 10.1093/gbe/evy039 29635328 PMC5892150

[ece35813-bib-0105] Zhan, L. , Paterson, I. G. , Fraser, B. A. , Watson, B. , Bradbury, I. R. , Nadukkalam Ravindran, P. , … Bentzen, P. (2017). MEGASAT: Automated inference of microsatellite genotypes from sequence data. Molecular Ecology Resources, 17(2), 247–256. 27333119 10.1111/1755-0998.12561

