# Supplementary material for: Evolutionary impacts differ between two exploited populations of northern bottlenose whale (Hyperoodon ampullatus)
Source: Ecol Evol. 2019 Nov 19;9(23):13567–84. doi: 10.1002/ece3.5813 (PMC6912904; doi:10.1002/ece3.5813)
Supplement: Supplementary file 1 [file ECE3-9-13567-s001.docx]

Appendix 1.

Table S1. *Hyperoodon ampullatus* microsatellite loci tested for consideration in population analyses, including DNA primer sequences and repeat motifs. Where appropriate the numbers and range of alleles per locus and Genbank accession number for northern bottlenose samples is included. The 37 new loci included in this study’s population structure analysis and the legacy loci (Dalebout et al., 2006) tested but not included (filtered) are indicated

| **Locus** | **Genbank** | **Fwd-Oligo** | **Rev-Oligo** | **No. alleles** | **Size** | **Motif** | **Analyses** | **Allelic richness** | ***H_o_*** | ***H_e_*** |
| --- | --- | --- | --- | --- | --- | --- | --- | --- | --- | --- |
| Hyam-135 | MH122619 | TTTCCAAAGCAAGATGAGAAGC | TCTGGGCTTGGGTGTGTTTC | 6 | 35-53 | GT | Retained | 5.95 | 0.4156 | 0.4193 |
| Hyam-133 | MH122618 | TGGTAGTAGTAGTGCATGCGTG | CCCAGCTCCCATTCCCTACAC | 2 | 35-37 | GT | Retained | 1.95 | 0.0065 | 0.0065 |
| Hyam-131 | MH122616 | AGCCCTGCTCAAGTAGACGG | ATGTATGTCCTGTGAGAACTGG | 3 | 32-38 | GT | Retained | 3 | 0.4805 | 0.5326 |
| Hyam-129 | MH122614 | GAGGATAGGCAGTCAGAGGG | CGCGGTGATGGGTCCTCTC | 2 | 37-39 | AG | Retained | 2 | 0.4675 | 0.4473 |
| Hyam-127 | MH122612 | AACCATCCTAAACTGGTCAGAC | TTGCAGTCAGTTCACCAATTG | 3 | 34-38 | AC | Retained | 3 | 0.5260 | 0.4707 |
| Hyam-126 | MH122611 | TTTAGCTTGCTGGGAGTGTG | TCCCAGGATAAAGCATGTGTG | 5 | 34-42 | AC | Retained | 5 | 0.7255 | 0.6698 |
| Hyam-124 | MH122610 | CATGGCTGGAGGGATGGG | TCATGGCCACACATAACTGGG | 3 | 31-37 | GCT | Retained | 3 | 0.3377 | 0.3580 |
| Hyam-122 | MH122609 | AGAAGCAGGTGTTCAGGTGTG | TCATCCATGCTCACCGTCCC | 6 | 35-47 | GT | Retained | 6 | 0.3856 | 0.3835 |
| Hyam-121 | MH122608 | TGAGAAGCAGAACCAACAGG | ATAAATCTCTTTGCGTGCGTG | 6 | 33-43 | GT | Retained | 6 | 0.7078 | 0.7039 |
| Hyam-120 | MH122607 | AGAGATAGAGGGACCCAGGCC | ACATTTGGCTGAAACTGGCTC | 3 | 33-37 | GT | Retained | 2.95 | 0.1753 | 0.1817 |
| Hyam-119 | MH122606 | GCGACTGGAAAGCAGAAATAG | TGGAGCAAGAAGGGAAGATTG | 3 | 32-36 | AC | Retained | 3 | 0.4091 | 0.4482 |
| Hyam-118 | MH122605 | TGGACACAGGGCTCCTCC | CACACAAAGATCCCAGGTCTGC | 2 | 33-35 | GT | Retained | 2 | 0.0779 | 0.0749 |
| Hyam-117 | MH122604 | GCGTGCTTGCGAATATGC | CTAAAGTTACACATGCGCTCAC | 3 | 30-34 | GT | Retained | 2.95 | 0.1104 | 0.1161 |
| Hyam-116 | MH122603 | GCATCTGTATTCATCAGTGCAG | ACTCCTTAAAGATAGCGTGCAC | 2 | 27-29 | GT | Retained | 2 | 0.0523 | 0.0509 |
| Hyam-114 | MH122602 | AGTGAACTGCTCTGATGTGTG | AGGGCAAGATATTCCTCCGC | 2 | 29-31 | GT | Retained | 2 | 0.2013 | 0.4888 |
| Hyam-113 | MH122601 | ACTTCCATCCTCCTATCTCCC | GCGTAGTGGTCTGTGGTC | 2 | 31-33 | CT | Retained | 2 | 0.0909 | 0.0868 |
| Hyam-109 | MH122600 | CAGGAGCTTAATGAGAAGTGGG | GTTCACAGGCTCTCACACTAG | 2 | 27-29 | GT | Retained | 2 | 0.1169 | 0.1214 |
| Hyam-108 | MH122599 | GCGCCCACATACTCTCATCTG | GGTTGCTGTGACTAGAGAGCAG | 2 | 27-31 | AC | Retained | 2 | 0.3137 | 0.4867 |
| Hyam-107 | MH122598 | TGGGAGGGAAGGTGAGAGCC | CTCTCACAGCCTGCCACATG | 2 | 28-30 | GT | Retained | 2 | 0.4238 | 0.4288 |
| Hyam-106 | MH122597 | GATATGAAGGAGGGCTGGC | TGCCCTCAGTGGTGACCC | 2 | 23-33 | GT | Retained | 2 | 0.4675 | 0.4857 |
| Hyam-102 | MH122594 | TGACTTTCTCCCTGCGTTGTG | ACAGAGACATGTTCACCGCCAC | 3 | 26-30 | GT | Retained | 3 | 0.3052 | 0.2986 |
| Hyam-099 | MH122592 | TTGGCCTCCGATGTTGGTTC | ATGCAATGCTAGAGGGAGGG | 2 | 29-31 | CT | Retained | 2 | 0.4610 | 0.4646 |
| Hyam-098 | MH122591 | AGAAATTGATGAACGGCGCGC | GTGAGGACAGCAAGCTCATTCC | 3 | 26-30 | AC | Retained | 3 | 0.2922 | 0.2705 |
| Hyam-095 | MH122590 | TGATGTGTGGGTTTGCATGCTC | CATACCACACACGCCGCG | 2 | 26-28 | GT | Retained | 2 | 0.4935 | 0.4857 |
| Hyam-094 | MH122589 | CCCTCCTTCAAGCGAGTG | TGTAGTGTGGGCAGAATGTG | 2 | 26-30 | AC | Retained | 2 | 0.5390 | 0.4983 |
| Hyam-040 | MH122587 | AGATGTATGTTTCCAGCAACCCAG | AACACAAGTGCAAAGCTTCTTCAG | 5 | 38-48 | AC | Retained | 5 | 0.4481 | 0.4263 |
| Hyam-034 | MH122586 | ATGTGTCCAAACTTATTTCTGCCC | AATTGGGAAAGACTAGGGAAGGAG | 4 | 33-39 | AC | Retained | 3.95 | 0.4026 | 0.3851 |
| Hyam-032 | MH122585 | ATCCACAGATGCCATGTCTCTC | TCAGTGGAAGGATATAGCCGAGTG | 4 | 26-40 | AC | Retained | 4 | 0.1169 | 0.1123 |
| Hyam-027 | MH122583 | GCTTTCTCTTTAGTCCTGGGTATGG | GCTTCTGCCCTGTCCATCTG | 4 | 35-41 | GT | Retained | 3.98 | 0.5867 | 0.6173 |
| Hyam-024 | MH122581 | GATACACATGATGGGAAGTCTGCG | TTCAGTGGAAGAGATGCAAACAC | 3 | 31-35 | AC | Retained | 3 | 0.5779 | 0.5376 |
| Hyam-016 | MH122579 | GTAGAAACCCGTGCCTTGGTG | TTCCATTTCCTCCAGCACGC | 3 | 35-39 | AC | Retained | 3 | 0.7013 | 0.6663 |
| Hyam-015 | MH122578 | CCAGACCTACAGCATCAGAAACGC | TACCTCCACCTCCACACACATG | 5 | 30-38 | GT | Retained | 4.97 | 0.6974 | 0.7257 |
| Hyam-012 | MH122577 | CAGAAATACACTGGGCGCAG | TTGCCCTCTCTTGTGTGCAC | 5 | 32-44 | GT | Retained | 5 | 0.6039 | 0.6270 |
| Hyam-008 | MH122576 | GTGACTGTACTCGCCTGGCC | GGGAACTGGAGTGTGAGAGG | 3 | 37-41 | GT | Retained | 3.91 | 0.3442 | 0.3647 |
| Hyam-007 | MH122575 | GGAACTGGGTAATTTGGCAGAGAGG | TCTCCTTCAGTCACGCCCTC | 3 | 28-34 | GT | Retained | 3 | 0.1373 | 0.1286 |
| Hyam-002 | MH122574 | GTGCAAGTGTGAGGGTGAGG | TTGTCAGGAATGATGGATGCTCAC | 8 | 19-46 | GT | Retained | 8 | 0.7347 | 0.7980 |
| Hyam-132 | MH122617 | TAAGAGTGACACCTGGCCAG | GCATGACTACTTTGGGATCTGG | 1 | 36 | CT | Filtered | 1 | 0 | 0 |
| Hyam-130 | MH122615 | AGGCGTCAGAGACCTTACAG | AGCTGCAACTTGTCCATATGG | 2 | 31-37 | CT | Filtered | 2 | 0.9542 | 0.4990 |
| Hyam-128 | MH122613 | AGACTGTCAGTTTGCCAGAG | CCCTCTAGGTGGCGTCTAAAG | 1 | 36 | AGG | Filtered | 1 | 0 | 0 |
| Hyam-104 | MH122596 | TGAGAAACCTTGAGAGTGGC | TTGACTCCAACACCATGACC | 1 | 29 | ATT | Filtered | 1 | 0 | 0 |
| Hyam-103 | MH122595 | CAGAGGTGTGCATGCTGAGTTG | ATCTGAACACCACCGGCCAG | 1 | 27 | GT | Filtered | 1 | 0 | 0 |
| Hyam-101 | MH122593 | ACGCCAGCCCAAACACAGTC | GGAATGTGCGGGTGTCAGGG | 1 | 29 | AC | Filtered | 1 | 0 | 0 |
| Hyam-093 | MH122588 | AACTTCTGCGTGCTTTGTCG | ACACACATCGATATTTGCACAC | 1 | 27 | GT | Filtered | 1 | 0 | 0 |
| Hyam-031 | MH122584 | CCGACTCCACTCCCGTAACACTC | CCCTACCCTCTACCCACCATG | 5 | 21-43 | AC | Filtered | 5.00 | 0.6068 | 0.6125 |
| Hyam-026 | MH122582 | GGGTCCAGAGCAAACGTGTG | GCCAGCCTCATGACTCTGTTG | 8 | 29-51 | GT | Filtered | 7.64 | 0.2119 | 0.3996 |
| Hyam-020 | MH122580 | CAACCTCTACACTCTGGCTTGCAC | CCAGTGGAGAAAGAAGGGCAGTTC | 1 | 32 | CT | Filtered | 1 | 0 | 0 |
| MK6 | AF237891 | GCTTATGGAGGTGTCAGCAG | TGTTGCAGCATCTCTCCTCC | 6 | 71-81 | GT | Filtered /Legacy | 5.76 | 0.5704 | 0.5610 |
| GT211 | AF309693 | TTGTCAATTCAAAGTAAAGGTTTTCC | GAGCCACTGCTCTATTCTATGAAAGC | 6 | 36-46 | GT | Filtered /Legacy | 6 | 0.6806 | 0.7077 |
